# Epidermal p65/NF-κB signalling is essential for skin carcinogenesis

**DOI:** 10.15252/emmm.201303541

**Published:** 2014-06-21

**Authors:** Chun Kim, Manolis Pasparakis

**Affiliations:** Institute for Genetics, Cologne Excellence Cluster on Cellular Stress Responses in Aging-Associated Diseases (CECAD), Centre for Molecular Medicine (CMMC), University of CologneCologne, Germany

**Keywords:** apoptosis, inflammation, mouse models of cancer, NF-κB signalling, skin carcinogenesis

## Abstract

The nuclear factor kappa B (NF-κB) signalling pathway exhibits both tumour-promoting and tumour-suppressing functions in different tissues and models of carcinogenesis. In particular in epidermal keratinocytes, NF-κB signalling was reported to exert primarily growth inhibitory and tumour-suppressing functions. Here, we show that mice with keratinocyte-restricted p65/RelA deficiency were resistant to 7, 12-dimethylbenz(a)anthracene (DMBA)-/12-O-tetra decanoylphorbol-13 acetate (TPA)-induced skin carcinogenesis. p65 deficiency sensitized epidermal keratinocytes to DNA damage-induced death *in vivo* and *in vitro*, suggesting that inhibition of p65-dependent prosurvival functions prevented tumour initiation by facilitating the elimination of cells carrying damaged DNA. In addition, lack of p65 strongly inhibited TPA-induced epidermal hyperplasia and skin inflammation by suppressing the expression of proinflammatory cytokines and chemokines by epidermal keratinocytes. Therefore, p65-dependent NF-κB signalling in keratinocytes promotes DMBA-/TPA-induced skin carcinogenesis by protecting keratinocytes from DNA damage-induced death and facilitating the establishment of a tumour-nurturing proinflammatory microenvironment.

## Introduction

Cancer development occurs in a multistep process that can be subdivided into tumour initiation, promotion and progression (Hanahan & Weinberg, 2011). Over the past years, inflammation has emerged as a crucial mediator of tumour growth and progression. In this view, immune and stromal cells in the tumour microenvironment are believed to support the proliferation and survival of neoplastic cells and to promote vessel growth and tumour cell motility (Mantovani *et al*, 2008). Of various signalling cascades involved in inflammatory processes, the NF-κB pathway has drawn extensive attention as a key mediator providing a link between inflammation and tumorigenesis (Karin, 2006). The NF-κB pathway is best known for its important role in regulating the expression of a large number of prosurvival and proinflammatory genes including Bcl-2 family members and several cytokines and chemokines (Pasparakis, 2009). In addition to its well-characterized function in inflammation and immunity, an increasing body of evidence supports that deregulation of NF-κB signalling both in tumour cells themselves and in stromal cells controls tumour growth.

In a mouse model of colitis-associated cancer, genetic deletion of IKK2/IKKβ, a kinase-activating canonical NF-κB signalling, in intestinal epithelial cells resulted in reduced tumour numbers by sensitizing carcinogen-exposed epithelial cells to apoptosis (Greten *et al*, 2004). In addition, inhibition of NF-κB signalling in mammary epithelial cells by expression of an IκBα super-repressor (IκBαSR) reduced tumour development in chemical medroxyprogesterone acetate (MPA)/7, 12-dimethylbenz(a)anthracene (DMBA)-dependent, and genetic PyVT- or ErbB2-driven models of mammary tumorigenesis (Pratt *et al*, 2009; Liu *et al*, 2010; Connelly *et al*, 2011). Moreover, NF-κB inhibition in lung epithelial cells by cell-specific ablation of IKK2 or p65/RelA or overexpression of IκBαSR significantly reduced tumour development *in vivo* in a Kras-driven model of lung adenocarcinoma (Meylan *et al*, 2009; Basseres *et al*, 2010; Xia *et al*, 2012). In keeping with those reports, studies in a chemical-induced lung tumour model also supported a protumorigenic role of NF-κB signalling in epithelial cells (Stathopoulos *et al*, 2007). Therefore, multiple studies in both chemical- and oncogene-driven models of carcinogenesis supported a protumorigenic role for NF-κB signalling in epithelial cells.

NF-κB signalling has also been shown to exhibit tumour-suppressing functions in different models. Inhibition of NF-κB signalling by ablation of IKK2 in hepatocytes resulted in strongly increased liver tumorigenesis induced by application of the chemical carcinogen diethylnitrosamine (DEN) (Maeda *et al*, 2005). Moreover, liver parenchymal cell-specific knockout of NEMO/IKKγ caused the spontaneous development of hepatocyte apoptosis, chronic hepatitis and hepatocellular carcinoma in mice, further supporting a tumour-suppressing function of IKK/NF-κB signalling in the liver (Luedde *et al*, 2007). Interestingly, in another model of liver carcinogenesis driven by biliary inflammation in Mdr2-deficient mice, inhibition of NF-κB by IκBαSR expression delayed the appearance of liver tumours (Pikarsky *et al*, 2004), suggesting that in hepatocytes, NF-κB can exert both tumour-promoting and tumour-suppressive functions. In contrast to other epithelial tissues such as the gut, mammary gland and lung where NF-κB signalling in epithelial cells exerts tumour-promoting functions, NF-κB signalling in epidermal keratinocytes was shown to oppose tumour development in the skin. Transgenic mice expressing IκBαSR in epidermal keratinocytes spontaneously developed squamous cell carcinomas (SCCs) that depended on TNF-mediated skin inflammation (van Hogerlinden *et al*, 1999; Lind *et al*, 2004). In addition, inhibition of NF-κB signalling in human keratinocytes promoted RAS-mediated transformation in a xenograft model (Dajee *et al*, 2003). Therefore, in sharp contrast to other epithelial cells, NF-κB is believed to exert tumour-suppressive functions in epidermal keratinocytes.

In order to elucidate the molecular and cellular mechanisms by which NF-κB mediates its tumour-suppressing functions in skin carcinogenesis, we studied the role of keratinocyte-intrinsic NF-κB signalling in a mouse model of skin carcinogenesis. Surprisingly, we found that mice with epidermal keratinocyte-specific deletion of p65 were resistant to DMBA-/TPA-induced skin tumorigenesis. Mechanistically, NF-κB inhibition in keratinocytes prevented tumour development by acting both during the initiation and promotion phases of skin carcinogenesis. Therefore, in contrast to its previously suggested tumour-suppressive role in the epidermis, our results show that p65-dependent NF-κB signalling exerts a tumour-promoting function in epidermal keratinocytes that is essential for DMBA-/TPA-induced skin carcinogenesis.

## Results

### Epidermal p65 is essential for skin tumorigenesis

**Figure 1:**
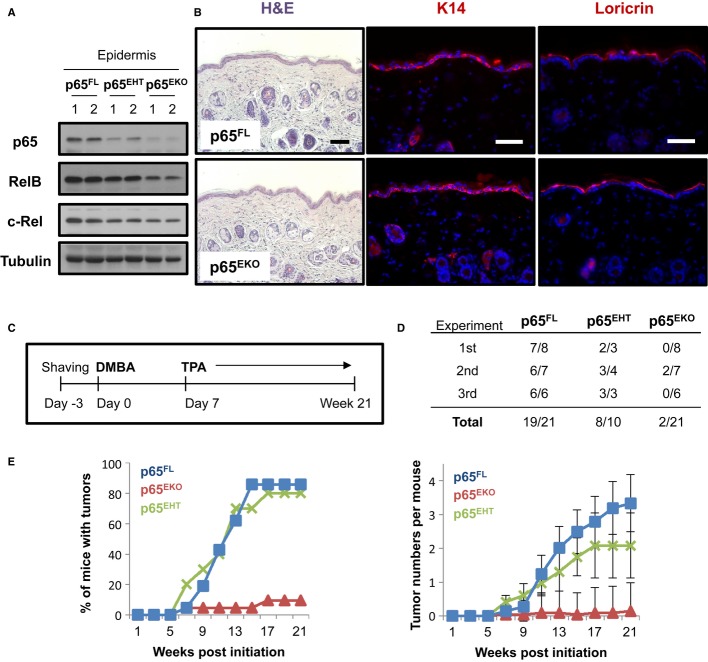
Mice with epidermal keratinocyte-restricted ablation of p65 are resistant to skin carcinogenesis Protein extracts prepared from epidermis obtained from tail skin of adult p65^FL^, p65^EHT^ and p65^EKO^ mice were analysed by immunoblotting with the indicated antibodies.Representative images of skin sections from adult p65^FL^ and p65^EKO^ mice stained with haematoxylin and eosin (H&E) or immunostained with antibodies against keratin 14 and loricrin (red). DNA was stained with DAPI (blue). Scale bar: 50 μm.Schematic depiction of the 7, 12-dimethylbenz(a)anthracene (DMBA)/12-O-tetra decanoylphorbol-13 acetate (TPA) skin carcinogenesis protocol used.Table showing tumour incidence from three independent experiments in groups of mice with the indicated genotypes. The number of animals showing tumours/the total number of animals per genotype are presented as counted at the end of the experiment.Tumour incidence presented as the percentage of mice with tumours (left panel) and mean tumour number per mouse (mean ± SEM) (right panel). Results from three independent experiments were pooled. p65^FL^ (*n* = 21), p65^EKO^ (*n* = 21) and p65^EHT^ (*n* = 10). Source data are available online for this figure.

We generated mice with specific deletion of the p65 gene in epidermal keratinocytes (p65^EKO^) by crossing mice carrying p65 floxed (p65^FL^) alleles (Luedde *et al*, 2008) with K14-Cre transgenic mice expressing Cre recombinase under the control of the keratin-14 (K14) promoter (Hafner *et al*, 2004). p65^EKO^ mice were born at Mendelian ratio and reached adulthood without showing apparent abnormalities. Immunoblot analysis of epidermal protein extracts showed that p65 was efficiently ablated in the epidermis of p65^EKO^ mice (Fig 1A). Interestingly, the loss of p65 resulted in decreased expression of the RelB and c-Rel NF-κB subunits, consistent with the notion that they are transcriptionally regulated by NF-κB (Hannink & Temin, 1990; Bren *et al*, 2001). Immunohistological analysis of skin sections from p65^EKO^ mice showed a normal epidermal–dermal organization and a normally differentiated stratified epidermis (Fig 1B). Therefore, as shown previously by Rebholz *et al* (2007) keratinocyte-specific p65 deficiency did not affect the development and differentiation of the epidermis, in contrast to a previous report that showed that skin grafts from p65-deficient embryos developed epidermal hyperplasia and suggested that p65 regulates normal epidermal proliferation and differentiation (Zhang *et al*, 2004).

To study the role of epidermal NF-κB signalling in skin carcinogenesis, we analysed the response of p65^EKO^, heterozygous p65^EHT^ and control p65^FL^ mice to a well-established model of two-stage chemical carcinogenesis. Tumour initiation was elicited by a single topical application of 100 nmol DMBA, and tumour promotion was induced by twice weekly topical treatment with 5 nmol 12-O-tetra decanoylphorbol-13 acetate (TPA) for up to 21 weeks (Fig 1C). Mice were macroscopically monitored for the development of papillomas during the course of the experiment and were sacrificed after week 21 when tissues were collected for further analysis. At the end of the regime, most p65^FL^ mice developed outwardly growing typical papillomas with an average of three tumours per mouse, consistent with previous reports showing that C57BL/6 mice are relatively resistant to DMBA-/TPA-induced skin tumorigenesis (Abel *et al*, 2009). In sharp contrast to their littermate control animals, p65^EKO^ mice were almost completely resistant to tumour formation (Fig 1D and E) under the same protocol, with only 2 out of 21 mice developing a small single papilloma each. p65^EHT^ mice developed papillomas similarly to p65^FL^ mice demonstrating that heterozygous loss of p65 in the epidermis was not sufficient to prevent tumour development (Fig 1D and E). This finding also shows that the resistance of p65^EKO^ mice to DMBA-/TPA-induced skin tumours cannot be attributed to Cre recombinase expression in keratinocytes. Therefore, p65-dependent NF-κB activation in epidermal keratinocytes is required for efficient DMBA-/TPA-induced skin tumorigenesis.

### NF-κB prevents DNA damage-induced cell death

**Figure 2:**
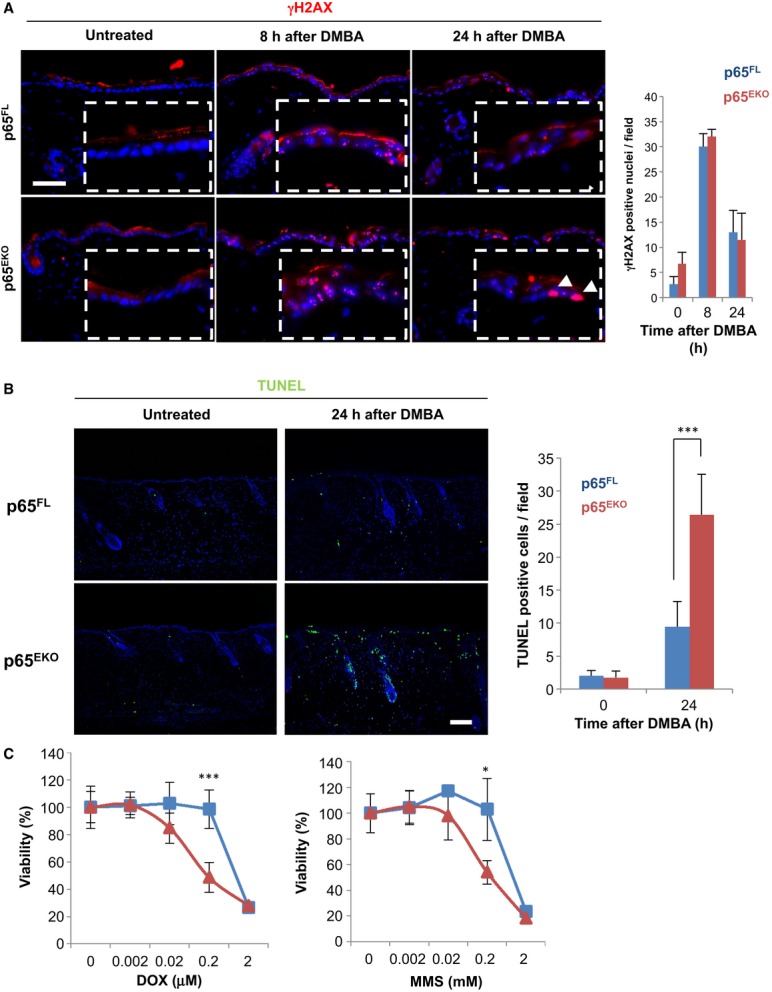
p65-deficient keratinocytes are sensitive to DNA damage-induced cell death Sections of dorsal skin obtained from mice with the indicated genotypes that were untreated or treated with 400 nmol of 7, 12-dimethylbenz(a)anthracene (DMBA) were immunostained against γH2AX. Pictures in dashed lined boxes represent magnified images of skin epidermis. Arrowheads indicate pan-nuclear staining of γH2AX. Data shown are representative of two independent experiments performed with 4–5 mice per group. The bar graph shows the average number of nuclei showing the typical punctate γH2AX-positive foci in the epidermis per optical field ± SD. Scale bar: 50 μm.TdT-mediated dUTP nick end labelling (TUNEL) staining of dorsal skin sections obtained from untreated or DMBA-treated mice with the indicated genotypes. The pictures are representative of two independent experiments carried with 4–5 mice per group. The bar graph shows the average number of TUNEL-positive cells per field ± SD. ***P = 0.0041. Scale bar: 50 μm.Primary keratinocytes from p65^FL^ and p65^EKO^ mice were treated with the indicated concentrations of doxorubicin (DOX) or methyl methanesulphonate (MMS), and cell viability was measured by neutral red uptake assay. The graphs are representative of three independent experiments. The values represent mean ± SD of 7 replicates. ***P = 0.0048; *P = 0.0217.

In order to understand the mechanisms by which p65 deficiency prevented tumour development in p65^EKO^ mice, we first analysed the effect of p65 loss on tumour initiation. During the first step of the DMBA/TPA skin tumorigenesis protocol, DMBA acts as a DNA mutagen in epidermal keratinocytes after being metabolized by resident skin Langerhans cells (LCs) (Modi *et al*, 2012). To rule out the possibility that the resistance of p65^EKO^ mice to tumour development could be a consequence of impaired DMBA metabolism, we investigated DNA damage responses (DDR) in the epidermis of p65^EKO^ and control mice by examining the phosphorylation of histone H2AX, γH2AX, which is generated in the vicinity of DNA damage lesions (Lukas *et al*, 2011). Immunostaining of skin sections with γH2AX-specific antibodies revealed that 8 h after a single topical application of 400 nmol of DMBA, epidermal cells in p65^EKO^ and their p65^FL^ littermates showed similar formation of well-defined γH2AX foci, indicating that metabolism of DMBA and activation of DDR are intact in p65^EKO^ mice (Fig 2A). In addition, immunohistochemical analysis of epidermal sheets revealed that keratinocyte-restricted p65 deficiency did not affect the numbers of Langerhans cells in the epidermis (Supplementary Fig S1). Interestingly, while most γH2AX foci began to disappear in the epidermis of the control animals at 24 h after DMBA treatment, the epidermis of p65^EKO^ mice frequently contained cells showing pan-nuclear staining of γH2AX at this time point (Fig 2A). Since nuclear panstaining of γH2AX has been reported to correlate with cell death in certain circumstances (Solier & Pommier, 2009; de Feraudy *et al*, 2010), this finding could indicate the presence of increased numbers of dying cells in the epidermis of p65^EKO^ mice. Indeed, TdT-mediated dUTP nick end labelling (TUNEL) staining revealed strongly increased numbers of dead keratinocytes in the skin of p65^EKO^ compared to p65^FL^ mice 24 h after a single topical application of 400 nmol (Fig 2B) or 100 nmol of DMBA (Supplementary Fig S2). To explore if the increased sensitivity of p65-deficient keratinocytes to DNA damage-induced death is DMBA specific, we tested the responses of primary keratinocytes derived from p65^EKO^ or p65^FL^ mice to treatment with other genotoxic agents. P65-deficient keratinocytes showed increased death compared to wild-type cells in response to treatment with doxorubicin (DOX), a DNA topoisomerase inhibitor, or methyl methanesulphonate (MMS), an alkylating agent (Fig 2C). Therefore, loss of p65 sensitized keratinocytes to DNA damage-induced cell death regardless of the mode of action of DNA damaging agents. Since the survival of mutation-bearing cells is critical for tumour initiation in chemically induced models of carcinogenesis, these results suggest that epidermal p65 deficiency protects mice from DMBA-/TPA-induced skin tumorigenesis, at least in part, by sensitizing keratinocytes to DNA damage-induced death.

### P65 deficiency does not alter DNA damage-induced p53 signalling in keratinocytes

**Figure 3:**
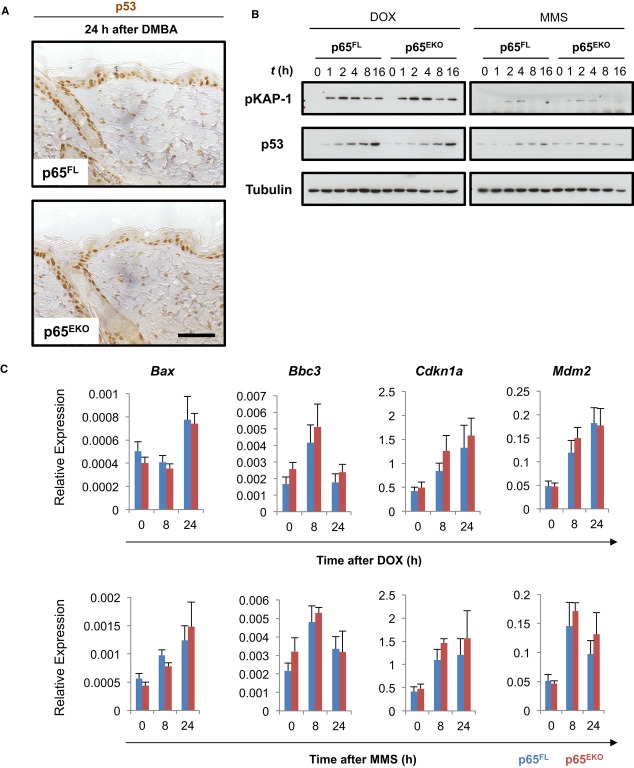
p65 deficiency does not affect DNA damage-induced p53 signalling in keratinocytes Immunohistochemical analysis of p53 expression in dorsal skin sections from p65^FL^ and p65^EKO^ mice treated with 400 nmol of 7, 12-dimethylbenz(a)anthracene (DMBA). Scale bar: 50 μm.Total cell extracts from 0.5 μM doxorubicin (DOX)- or 0.5 mM methyl methanesulphonate (MMS)-treated primary keratinocytes were analysed with immunoblotting with the indicated antibodies. Data shown are representative of two independent experiments.Real-time qPCR analysis of p53 target gene expression in keratinocytes at different time points after 0.5 μM DOX or 0.5 mM MMS treatment. Expression levels are presented relative to that of the ‘housekeeping’ gene *Ppia* (mean ± SD of triplicates). Data are representative of three independent experiments. Source data are available online for this figure.

In response to DNA damage, the tumour suppressor protein p53 is stabilized leading to cell cycle arrest, senescence and cell death (Meek, 2009). Since previous studies suggested that the NF-κB and p53 pathways interact (Ryan *et al*, 2000; Tergaonkar *et al*, 2002), we sought to determine whether p65 deficiency affected DNA damage-induced activation of p53 in keratinocytes. Immunostaining of skin sections obtained from mice 24 h after application of 400 nmol (Fig 3A) or 100 nmol (Supplementary Fig S3) of DMBA failed to reveal differences in p53 stabilization in the epidermis of p65^EKO^ and control mice, suggesting that p65 deficiency did not affect p53 activation. Of note, immunohistochemical assessment of p53 expression in the skin of untreated mice revealed that, in contrast to p65^FL^ mice that did not show p53 staining, the epidermis of p65^EKO^ mice contained a small number of keratinocytes showing nuclear p53 staining (Supplementary Fig S3). This seems to be the effect of DNA damage induced by Cre recombinase expression rather than p65 ablation, since K14cre animals not carrying p65 floxed alleles also showed a similar staining pattern (Supplementary Fig S3). Nevertheless, our results showing that mice with heterozygous epidermis-specific knockout of p65 (p65^EHT^) developed skin tumours similarly to p65^FL^ mice after DMBA/TPA treatment (Fig 1D and E) demonstrate that this low level DNA damage induced by Cre recombinase did not have a measurable effect in DMBA-/TPA-induced skin tumorigenesis.

To further address the cell-intrinsic role of p65 in the DDR, we also examined the activation of the DDR in primary keratinocytes from p65^EKO^ and control mice after treatment with DOX or MMS. In response to DNA damage, p65-deficient and wild-type keratinocytes showed similar kinetics of KAP-1 phosphorylation, a marker of DNA damage (White *et al*, 2006), indicating that the absence of p65 did not affect the initiation of the DDR (Fig 3B). In addition, the absence of p65 did not alter the kinetics of DNA damage-induced p53 protein stabilization (Fig 3B) or p53 mRNA expression (Supplementary Fig S4) in primary keratinocytes consistent with our *in vivo* findings. To further assess the activation of p53, we measured the expression of a number of classical p53 target genes in keratinocytes treated with DOX or MMS. qRT-PCR analysis of *Bax, Bbc3, Cdkn1a, MDM2, Bid and Bak1* mRNA levels did not reveal differences between p65-deficient and wild-type keratinocytes, providing further evidence that the absence of p65 did not affect the p53 response after DNA damage in keratinocytes (Fig 3C and Supplementary Fig S4). Collectively, these results showed that the increased sensitivity of p65-deficient keratinocytes to DNA damage-induced death is not due to an altered activation of the p53 pathway.

### Reduced cIAP2 expression in p65-deficient keratinocytes

**Figure 4:**
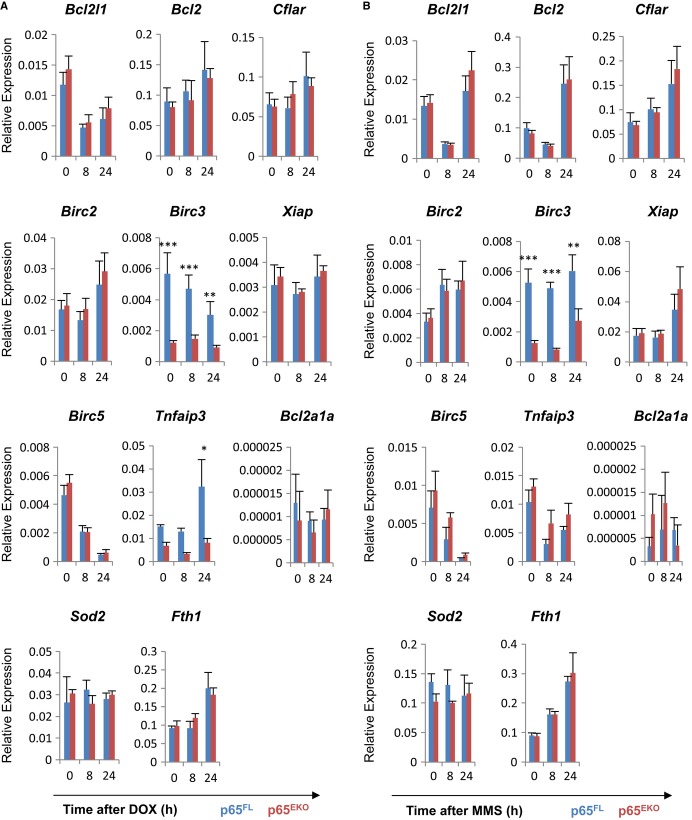
p65-deficient keratinocytes show reduced expression of *Birc3* A, B qRT-PCR expression analysis of survival genes in wild-type and p65-deficient primary mouse keratinocytes treated with 0.5 μM doxorubicin (DOX) (A) or 0.5 mM methyl methanesulphonate (MMS) (B). Expression levels are presented relative to that of the ‘housekeeping’ gene *Ppia* (mean ± SD of triplicates of two biologically different samples per genotype in each experimental group). Data are representative of three independent experiments. (A) *Birc3* (At 0, 8 and 24 h, ***P = 0.0005*,* ***P = 0.0021 *and* **P = 0.0086*,* respectively); *Tnfaip3* (At 24 h, *P = 0.0186*)*. (B) *Birc3* (At 0, 8 and 24 h, ***P = 0.0001*,* ***P = 0.0043 *and* **P = 0.0094*,* respectively).

The activation of NF-κB protects cells from cytotoxic stress by regulating the expression of genes promoting cell survival (Baldwin, 2012). We therefore examined whether the absence of p65 resulted in impaired DNA damage-induced expression of a number of classical NF-κB-dependent survival genes. Unexpectedly, we were not able to detect significant down-regulation of most NF-κB-dependent survival genes in DOX- or MMS-treated p65-deficient keratinocytes compared to similarly treated wild-type cells (Fig 4). Nevertheless, we found that the basal expression level of *Birc3*, the gene encoding the anti-apoptotic protein cIAP2 that has been implicated in cell survival in response to DNA damage (Tenev *et al*, 2011), was strongly impaired in p65^EKO^ keratinocytes suggesting that the transcription of *Birc3* is p65 dependent (Fig 4). Interestingly, the expression of other *Birc* gene family members including *Birc2* and *Xiap* was not affected in the absence of p65. This result is consistent with previous studies reporting that *Birc3* is a p65-specific transcriptional target (Chen *et al*, 2003; Zhao *et al*, 2011). In addition, we found that the *Tnfaip3* gene encoding A20 was down-regulated in p65-deficient keratinocytes in response to DOX, but not to MMS (Fig 4). These results indicate that impaired expression of cIAP2 and perhaps other prosurvival proteins such as A20 could contribute to the increased susceptibility of p65-deficient keratinocytes to DNA damage-induced death.

### Epidermal p65 is indispensable for TPA-induced skin inflammation and keratinocyte proliferation

**Figure 5:**
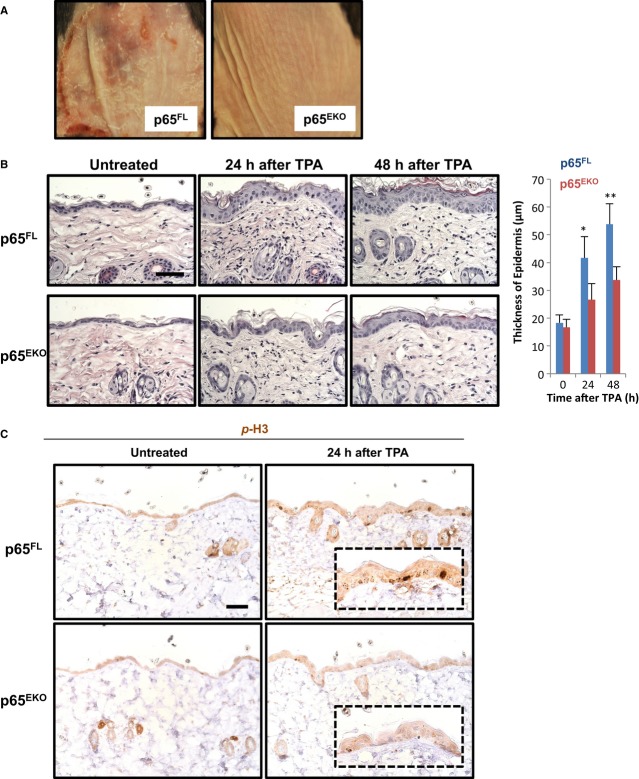
12-O-tetra decanoylphorbol-13 acetate (TPA)-induced epidermal hyperplasia is impaired in p65^EKO^ mice Representative macroscopic images of dorsal skin of p65^FL^ and p65^EKO^ mice 48 h after a single topical application of a high dose (25 nmol) of TPA. Three mice per group were used in the experiment.H&E staining of dorsal skin from p65^FL^ and p65^EKO^ mice that were untreated or treated with 5 nmol of TPA. Pictures shown are representative of two independent experiments performed with 4–5 animals per group. The bar graph shows the average epidermal thickness of animals in each group ± SD. *P = 0.039; ***P = 0.0041. Scale bar: 50 μm.Sections from dorsal skin of p65^FL^ and p65^EKO^ mice that were untreated or treated with 5 nmol of TPA were analysed by immunohistochemistry for phosphorylated histone 3. Data shown are representative of two independent experiments performed with 4–5 animals per group. Scale bar: 50 μm.

In the DMBA-/TPA-induced model of skin carcinogenesis, repeated applications of TPA are required for tumour promotion after DMBA-induced tumour initiation. TPA induces skin inflammation and epidermal hyperplasia providing a permissive microenvironment for the survival and expansion of keratinocytes carrying DNA mutations during the early stages and ultimately tumour growth. Because NF-κB is also known to participate in inflammation and cell cycle control (Karin, 2006; Pasparakis, 2009; Baldwin, 2012), we hypothesized that p65 might act in keratinocytes to regulate TPA-induced tumour promotion and examined whether epidermis-specific p65 deficiency affected TPA-induced inflammatory epidermal hyperplasia. We first analysed the response of p65^EKO^ and their p65^FL^ littermates to a single topical application of a high dose (25 nmol) of TPA. In p65^FL^ mice, TPA elicited a strong inflammatory response in the skin, macroscopically visible with a high level of erythema, scaling and some focal erosion 48 h after treatment. In sharp contrast, the skin of p65^EKO^ animals did not show any sign of irritation after similar TPA treatment (Fig 5A). It should be noted that the dose of TPA we applied in this experiment was 5 times higher than the dose we used for the repeated TPA treatments during the carcinogenesis protocol. We therefore proceeded with a more detailed analysis of the response of the skin to 5 nmol TPA, which is the dose applied repeatedly during the tumour promotion phase. A single application of 5 nmol TPA did not induce macroscopically visible skin inflammation in p65^FL^ mice. However, histological analysis of skin sections revealed strongly increased epidermal thickness and dermal cellularity in p65^FL^ mice 24 and 48 h after TPA treatment. In contrast, TPA treatment induced only mild epidermal thickening in p65^EKO^ mice (Fig 5B). Immunostaining of skin sections with antibodies recognizing phosphorylated histone-3, a mitotic marker, revealed strongly increased keratinocyte proliferation in the epidermis of p65^FL^ mice compared to p65^EKO^ animals (Fig 5C), demonstrating that the p65-deficient epidermis failed to elicit a strong hyperplastic response upon TPA treatment.

**Figure 6:**
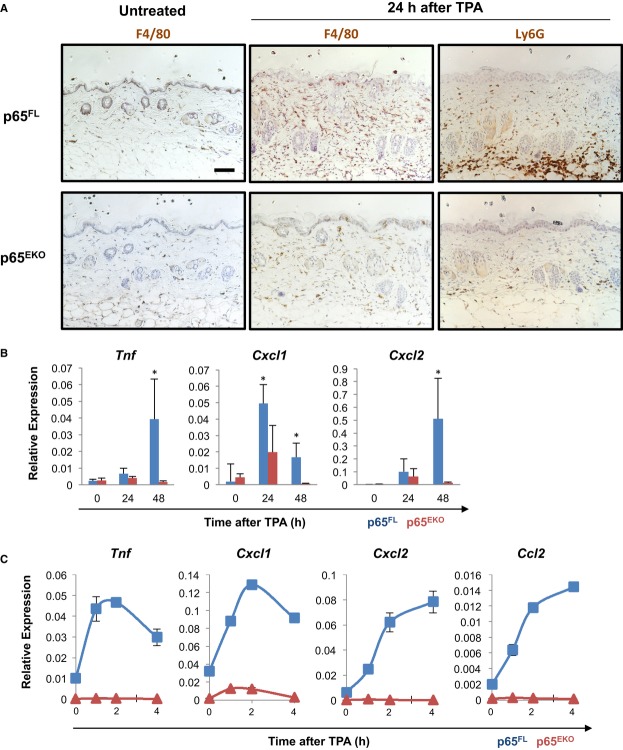
12-O-tetra decanoylphorbol-13 acetate (TPA)-induced inflammatory responses are attenuated in p65^EKO^ mice Skin sections from p65^FL^ and p65^EKO^ mice that were untreated or treated with 5 nmol of TPA were immunostained with antibodies against F4/80 or Ly6G. Data shown are representative of two independent experiments with 4–5 animals per group. Scale bar: 50 μm.Quantitative real-time PCR analysis of the expression of *TNF, Cxcl1* and *Cxcl2* mRNA in skin tissues from p65^FL^ and p65^EKO^ mice that were untreated or treated with 5 nmol of TPA (3 mice per group). Expression levels are presented relative to that of the ‘housekeeping’ internal control gene *Ppia* (mean ± SD). Data are representative of two independent experiments. *Tnf* (*P = 0.0321); *Cxcl1* (*P = 0.0487 at 24 h, *P = 0.0209 at 48 h); *Cxcl2* (*P = 0.0356).Quantitative real-time PCR analysis of the expression of *TNF*, *Cxcl1*, *Cxcl2* and *Ccl2* mRNA in primary keratinocytes from p65^FL^ and p65^EKO^ mice before and after treatment with 100 nM TPA. Expression levels are presented relative to that of *Ppia* (mean ± SD. of triplicates of two biologically different samples per genotype in each experimental group).
Data are representative of two independent experiments.

To examine the induction of skin inflammation by TPA, we first assessed the presence of myeloid cells in the skin of p65^FL^ and p65^EKO^ mice before and after TPA treatment. Untreated p65^FL^ and p65^EKO^ mice had similar numbers of skin-resident F4/80-positive macrophages suggesting that epidermal p65 deficiency did not affect basal immune homoeostasis in the skin. A single application of 5 nmol TPA induced strong infiltration of macrophages (F4/80 positive) and granulocytes (Ly6G positive) in the skin of p65^FL^ mice (Fig 6A). In contrast, the skin of similarly treated p65^EKO^ mice contained substantially reduced numbers of macrophages and granulocytes compared to p65^FL^ mice (Fig 6A), demonstrating that epidermal p65 deficiency largely prevented the TPA-induced recruitment of myeloid cells in the skin. Furthermore, the skin of p65^EKO^ mice showed strongly reduced expression of *Cxcl1/2,* two chemokines that are important for the recruitment of granulocytes (Lazennec & Richmond, 2010), as well as TNF, a cytokine previously shown to regulate DMBA-/TPA-induced skin carcinogenesis (Moore *et al*, 1999; Suganuma *et al*, 1999), after TPA treatment compared to the skin of p65^FL^ mice (Fig 6B). The impaired expression of TNF and Cxcl1/2 in the skin of p65^EKO^ mice could be due to reduced expression of these mediators in both epidermal and other dermal and immune cell populations. In order to specifically address the keratinocyte-intrinsic function of p65 in regulating TPA-induced inflammatory gene expression, we used cultured primary epidermal keratinocytes. TPA stimulation induced strong expression of *Tnf*, *Cxcl1/2* and *Ccl2* in wild-type but not in p65-deficient primary keratinocytes demonstrating that the TPA-induced expression of these inflammatory mediators in keratinocytes requires p65 (Fig 6C). Therefore, epidermal keratinocyte-specific ablation of p65 inhibited the TPA-induced expression of chemokines and cytokines and the tumour-promoting inflammatory hyperplastic response in the skin.

## Discussion

The role of NF-κB in tumour development is complex, exhibiting both tumour-promoting and tumour-suppressive properties. In the skin, NF-κB signalling in epidermal keratinocytes has been suggested to perform primarily tumour-suppressing functions. Our results presented here contrast with this notion and show that p65-dependent NF-κB signalling in epidermal keratinocytes promotes skin tumour formation in the DMBA/TPA model of two-stage skin carcinogenesis. Mechanistically, we found that epidermal p65 deficiency affected both DMBA-induced tumour initiation and TPA-induced tumour promotion. p65 deficiency sensitized keratinocytes to DNA damage-induced death *in vivo* and *in vitro*, suggesting that inhibition of NF-κB prevented tumour initiation by facilitating the clearance of cells bearing damaged DNA. Furthermore, p65 deficiency prevented TPA-induced expression of proinflammatory cytokines and chemokines by epidermal keratinocytes *in vivo* and *in vitro*, resulting in impaired recruitment of inflammatory cells to the skin. Keratinocyte-specific p65 deficiency also prevented TPA-induced epidermal hyperplasia. Therefore, p65-mediated NF-κB activation in keratinocytes is also required for efficient TPA-mediated tumour promotion by regulating the release of cytokines and chemokines that induce skin inflammation and epidermal hyperplasia.

Our results support a tumour-promoting role of epidermal p65/NF-κB signalling, in contrast with previous studies, suggesting that NF-κB displays primarily tumour-suppressive functions in keratinocytes. Dajee *et al* showed that expression of an IκBα super-repressor mutant (IκBαSR) prevented growth arrest induced by oncogenic Ras in primary human keratinocytes resulting in the formation of SCCs upon transplantation into immunodeficient mice. Importantly, keratinocytes expressing IκBαSR or Ras alone failed to establish tumours, suggesting that NF-κB inhibition by itself was not sufficient to trigger tumorigenesis but synergized with oncogenic Ras to support neoplastic transformation. Since tumours induced in the DMBA/TPA model of skin carcinogenesis usually Ras mutations, it is interesting that IκBαSR expression in the study by Dajee *et al* had opposing effects in Ras-mediated tumorigenesis compared to our studies using epidermal p65 deficiency. These contradicting results could be explained by the different nature of the two models. In the DMBA/TPA model, Ras mutations are induced by a chemical carcinogen causing DNA damage followed by an inflammation-dependent phase of tumour promotion, while in the experiments performed by Dajee *et al,* oncogenic Ras was delivered to keratinocytes by retroviral expression vectors. We cannot exclude that when tumour initiation and promotion are bypassed by overexpression of oncogenic Ras, then NF-κB displays tumour-suppressing properties. Another difference that might have contributed to the different outcome in the two models is that the tumorigenic capacity of human keratinocytes expressing IκBαSR and oncogenic Ras was assessed by transplantation into immunodeficient mice, while in our experiments, the mice were immune competent. Furthermore, considering the complexity of the NF-κB signalling cascade, it is also possible that while both IκBαSR expression and p65 deficiency are generally and widely accepted means to inhibit NF-κB-dependent gene expression, they might induce qualitatively different effects on NF-κB activation that differentially affect epidermal tumorigenesis. For example, the composition of NF-κB dimers in p65-deficient cells is different from wild-type cells, including an increased nuclear accumulation of p50 homodimers (Luedde *et al*, 2008), which may further potentiate inhibition of NF-κB-dependent gene expression by binding to κB sites and suppressing the expression of specific target genes. In addition, IκBα does not bind all NF-κB dimers with equal affinity therefore overexpression of IκBαSR is also likely to have complex effects on NF-κB dimer availability and gene expression regulation that are still poorly understood (Oeckinghaus & Ghosh, 2009). Moreover, IκBαSR overexpression could also potentially mediate additional NF-κB-independent functions. For example, IκBα has been reported to act in the nucleus to directly repress the expression of Notch genes by associating with nuclear co-repressors and histone acetyltransferases and deacetylases (Aguilera *et al*, 2004). Since Notch inhibition in keratinocytes promoted epidermal tumorigenesis (Demehri *et al*, 2009), IκBαSR overexpression in the epidermis could potentially enhance tumour development by interfering with the expression of Notch target genes. In addition, a recent study showed that chromatin-bound IκBα regulates a subset of Polycomb target genes controlling differentiation and cancer in keratinocytes (Mulero *et al*, 2013), further supporting an NF-κB-independent function of IκBα in skin carcinogenesis. Taken together, the opposing results obtained in our study compared to the work of Dajee *et al* likely reflect the complexity of the NF-κB signalling system and its regulators and suggest that more studies are needed in order to fully understand the multifaceted role of this pathway in cancer development.

In another study, transgenic mice expressing IκBαSR in epidermal keratinocytes developed spontaneous skin tumours in the FVB/N genetic background (van Hogerlinden *et al*, 1999). However, in this case, tumour development was preceded by skin inflammation and epidermal hyperplasia that depended on TNF signalling as the skin lesions did not develop when the mice were crossed into the TNFR1-deficient genetic background (Lind *et al*, 2004). We previously showed that mice lacking IKK2 or NEMO specifically in epidermal keratinocytes (IKK2^EKO^ and NEMO^EKO^, respectively) spontaneously developed severe TNFR1-dependent inflammatory skin lesions and died about 10 days after birth (Pasparakis *et al*, 2002; Nenci *et al*, 2006). Although knockout of IKK subunits may also induce NF-κB-independent effects, the findings that both IκBαSR overexpression and ablation of IKK2 or NEMO in the epidermis resulted in skin inflammation demonstrate that epidermal NF-κB signalling regulates skin immune homoeostasis. The development of skin tumours in the K5-IκBαSR transgenic mice is likely the consequence of the strong inflammatory response induced by the disturbance of homoeostatic NF-κB signalling in the epidermis and the high susceptibility of the FVB/N strain to tumour development (Hennings *et al*, 1993).

Although our findings contrast with previous reports suggesting a tumour-suppressing role of NF-κB in the epidermis, they are consistent with a number of other studies suggesting a tumour-promoting role for NF-κB in epithelial tissues. NF-κB has been suggested to support oncogenic RAS-mediated tumorigenesis in fibroblasts, and different epithelial cells by providing survival and proliferation signals (Mayo *et al*, 1997; Meylan *et al*, 2009; Basseres *et al*, 2010; Xia *et al*, 2012). In addition, one recent study showed that in keratinocytes, oncogenic Ras-induced cell dedifferentiation and proinflammatory gene expression are largely dependent on NF-κB (Cataisson *et al*, 2012). Taken together, our results demonstrate that p65-dependent NF-κB activation in epidermal keratinocytes promotes inflammation-associated tumour development by regulating the survival of cells exposed to DNA damage and the establishment of a tumour-promoting inflammatory microenvironment.

## Materials and Methods

### Mice and skin carcinogenesis

Mice with loxP-flanked p65 alleles were previously described (Luedde *et al*, 2008). At 7–9 weeks of age, a patch of dorsal hair from each mouse was removed using an electric hair clipper (ER121; Panasonic) and a fine electric shaver (ES 7036; Panasonic). Three days after hair removal, a single initiation dose (100 nmol) of DMBA (D3254; Sigma) in 100 μl acetone (A1600; Applichem) was applied to the shaved area using a brush. Starting 1 week after DMBA application, the DMBA-initiated area was treated twice weekly with 5 nmol of TPA (P1585; Sigma) in 100 μl acetone. The appearance of tumours was monitored and recorded every 2 weeks. Papillomas that were bigger than a diameter of 1 mm and were present for longer than 2 weeks were counted. The tumour incidence and the number and the size of tumours per mouse were examined for 21 weeks. All animal procedures were conducted in accordance with European, national and institutional guidelines and protocols and were approved by local government authorities (Landesamt für Natur, Umwelt und Verbraucherschutz Nordrhein-Westfalen, Germany).

### Single DMBA or TPA topical treatment

Hairs on the back skin of 7- to 9-week-old animals were shaved 3 days before a DMBA or TPA single treatment. Four hundred nmol of DMBA in acetone or either 5 or 25 nmol of TPA in acetone was applied to the shaved back skin of mouse as described in the main text.

### Histological analysis

Skin samples were embedded in paraffin and cut in 5-μm sections. In order to evaluate basic histopathological features, the sections were stained with haematoxylin and eosin and examined under a microscope. Several immunohistochemical markers were examined at different time points as described in the figure legends. The following antibodies were used: K14 (MS115; Thermoscientific), Loricrin (PRB-145P; Covance), γH2AX (05-636; Millipore), p53 (NCL-p53-CM5p; Leica), Phospho-Histone H3 (9701; Cell signalling), F4/80 (MCAP497; Serotec), Ly6G (551459; BD), anti-Mouse IgG-Alexa594 (A11005; Invitrogen), anti-Rabbit IgG-Alexa594 (A11012; Invitrogen), anti-Rabbit IgG-Biotin (NEF813; Perkin Elmer), anti-Mouse IgG-Biotin (BA-9200; Biozol) and anti-Rat IgG-Biotin (E0468; Dako). TdT-mediated dUTP nick end labelling assay was performed using DeadEnd™ Fluorometric TUNEL System (G3250; Promega).

### Primary keratinocyte culture

Keratinocytes were obtained from 2- to 3-day-old mice as described (Lichti *et al*, 2008) with a minor modification. In brief, the epidermis was separated from the dermis by incubation in 0.25% trypsin at 4°C overnight. The isolated keratinocytes from the epidermal sheet were cultured in medium composed of Eagle's Minimum Essential Medium (EMEM) (06-174G; Lonza), 10 mM HEPES (15630; Gibco), Antibiotic-Antimycotic (15240; Gibco), 50 μM CaCl_2_, 4% chelex-treated fetal calf serum (FCS) and 10 ng/ml epidermal growth factor (EGF). Collagen-coated tissue culture dishes were purchased from BD science.

### Immunoblot analysis

Protein extracts were separated on SDS–PAGE and were transferred to a PVDF membrane (IPVH00010; Millipore). SuperSignal® West Pico Chemiluminescent substrate (34080; Thermo) was used to visualize the signal. The membranes were reprobed after incubation in stripping buffer (21059; Thermo). Antibodies against the following proteins were used: p65 (sc-372; Santacruz), RelB (4922; Cell signaling), c-Rel (sc-71; Santacruz), Tubulin (T6074; Sigma), Phospho-KAP-1 (A300-767A; Bethyl) and p53 (2524; Cell signalling).

### Cell death assay

Primary keratinocytes were seeded in collagen-coated 96-well plates. Seventy-two hours after incubation with indicated amounts of DOX (D1515; Sigma) or MMS (129925; Sigma), cell viability was measured by neutral red assay as described (Repetto *et al*, 2008).

### Gene expression analysis

Total RNA from skin tissues or keratinocytes treated with DOX or MMS was purified using TRIzol (15596018; Invitrogen) and RNA spin column (740955; Macherey-Nagel). The synthesized cDNA (18080-051; Invitrogen) was added to PCR master mix (4367659; Applied biosystems), and quantitative real-time PCR was performed in a PCR machine (7900HT; Applied biosystems).

### Statistics

Statistical analyses were performed with unpaired Student's *t*-test.

## Supporting Information

Supplementary Figure S4

Review Process File
