# Supplementary material for: Epidermal p65/NF-κB signalling is essential for skin carcinogenesis
Source: EMBO Mol Med. 2014 Jun 21;6(7):970–83. doi: 10.15252/emmm.201303541 (PMC4119358; doi:10.15252/emmm.201303541)
Supplement: Supplementary file 1 — Supplementary Figure S1 [file emmm0006-0970-SD1.pdf]

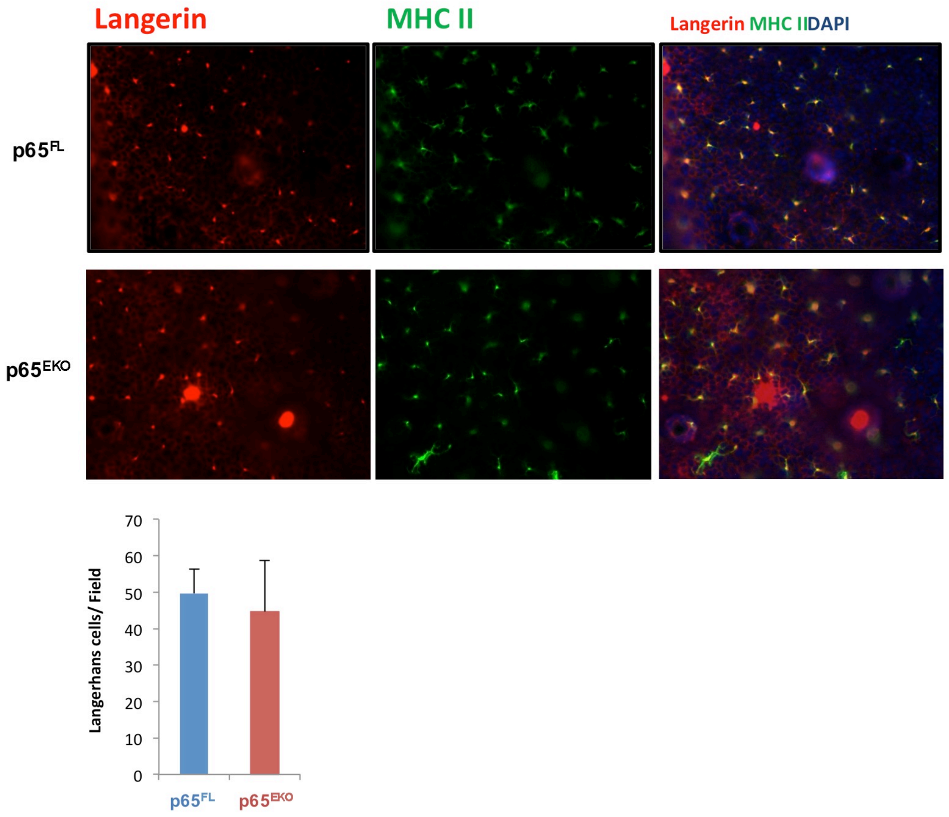

Figure S1. Keratinocyte p65 deficiency does not affect the number of Langerhans cells in the epidermis.

Ear epidermal sheets from p65^{FL} and p65^{EKO} mice were immunostained with antibodies against Langerin and MHCII and stained with DAPI to visualise nuclei. The graph shows the average number of Langerin and MHC II double positive cells per field. The experiment was performed with 3 mice per group.
