# Supplementary material for: Epidermal p65/NF-κB signalling is essential for skin carcinogenesis
Source: EMBO Mol Med. 2014 Jun 21;6(7):970–83. doi: 10.15252/emmm.201303541 (PMC4119358; doi:10.15252/emmm.201303541)
Supplement: Supplementary file 2 — Supplementary Figure S2 [file emmm0006-0970-SD2.pdf]

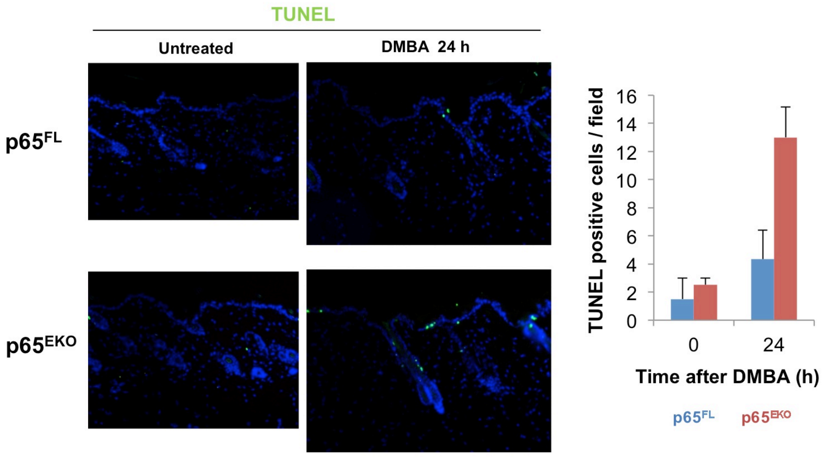

Figure S2. p65 deficiency sensitizes keratinocytes to DMBA-induced apoptosis.

Skin sections from mice that were untreated or treated with 100 nmol of DMBA were stained for TUNEL. The graph shows the average number of TUNEL positive cells per field. The experiment was performed with 3-4 mice per group.
