# Supplementary material for: Epidermal p65/NF-κB signalling is essential for skin carcinogenesis
Source: EMBO Mol Med. 2014 Jun 21;6(7):970–83. doi: 10.15252/emmm.201303541 (PMC4119358; doi:10.15252/emmm.201303541)
Supplement: Supplementary file 3 — Supplementary Figure S3 [file emmm0006-0970-SD3.pdf]

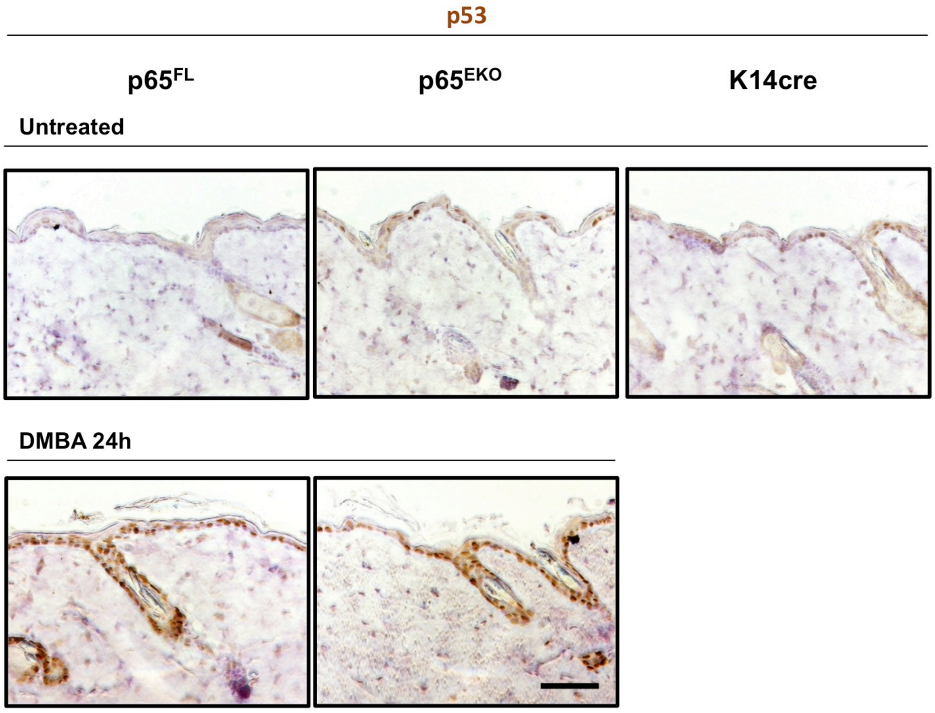

Figure S3. p65 deficiency does not alter p53 expression in keratinocytes after DNA damage.

Immunohistochemical analysis of p53 expression in skin sections from $p65^{FL}$, $p65^{EKO}$, and K14-Cre mice that were untreated or 24h after application of 100 nmol of DMBA. The experiment was performed with 3-4 mice per group. Scale bar: 50 µm.
