# Supplementary material for: Epidermal p65/NF-κB signalling is essential for skin carcinogenesis
Source: EMBO Mol Med. 2014 Jun 21;6(7):970–83. doi: 10.15252/emmm.201303541 (PMC4119358; doi:10.15252/emmm.201303541)
Supplement: Supplementary file 4 — Supplementary Figure S4 [file emmm0006-0970-SD4.pdf]

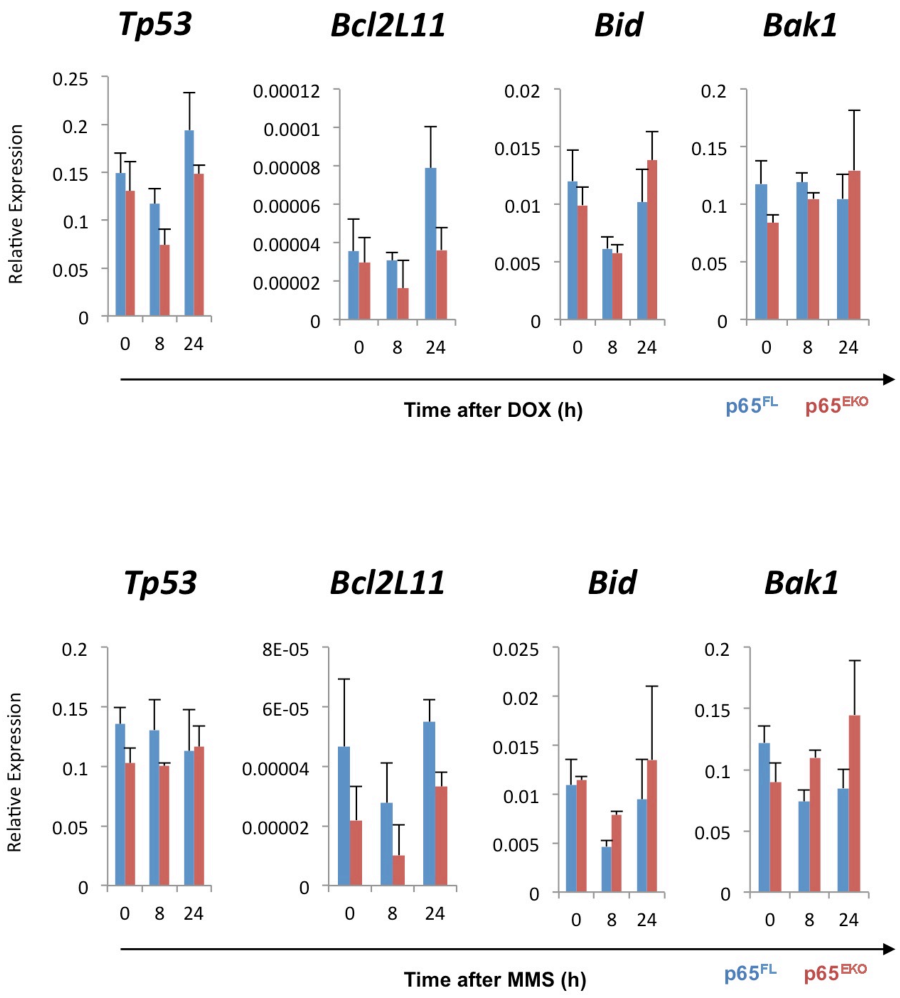

Figure S4. p65-deficiency does not affect p53-dependent gene expression.

qRT-PCR expression analysis of genes in DOX- or MMS- treated primary keratinocytes. Expression levels are presented relative to that of the 'housekeeping' gene Ppia (mean ± sd of triplicates).
