# Supplementary figures and images for: Epidermal p65/NF-κB signalling is essential for skin carcinogenesis
Source: EMBO Mol Med. 2014 Jun 21;6(7):970–83. doi: 10.15252/emmm.201303541 (PMC4119358; doi:10.15252/emmm.201303541)

Figure 1A

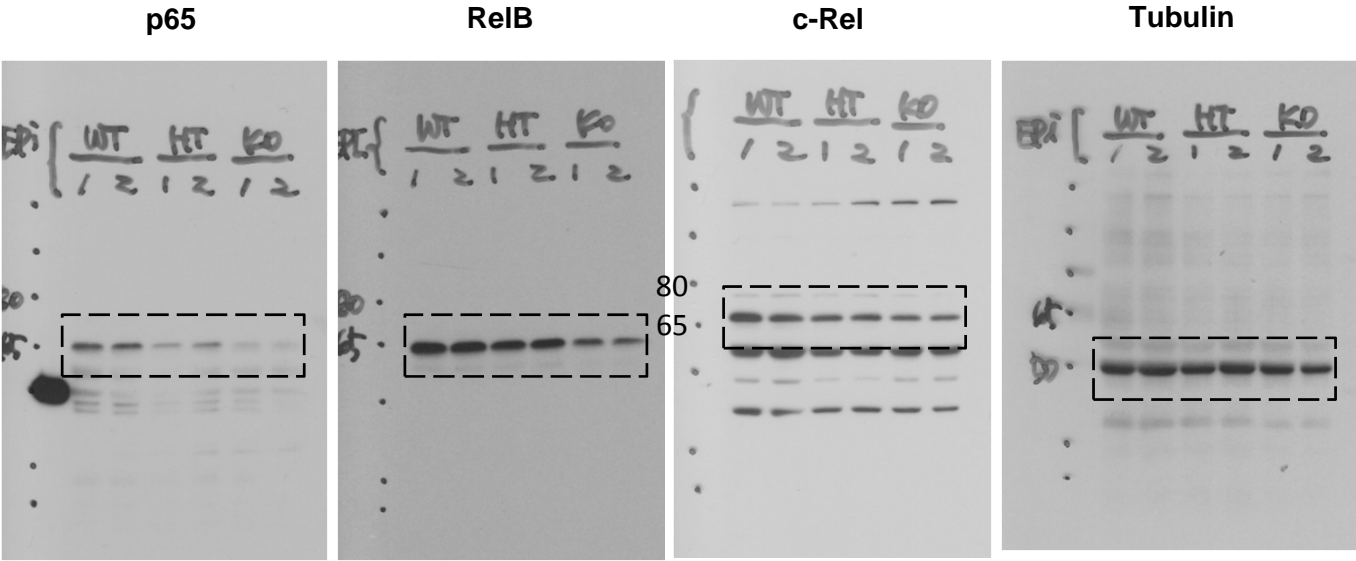

Supplement: Supplementary file 6 — Source Data for Figure 1 A [file emmm0006-0970-SD6.pdf]

Figure 3B

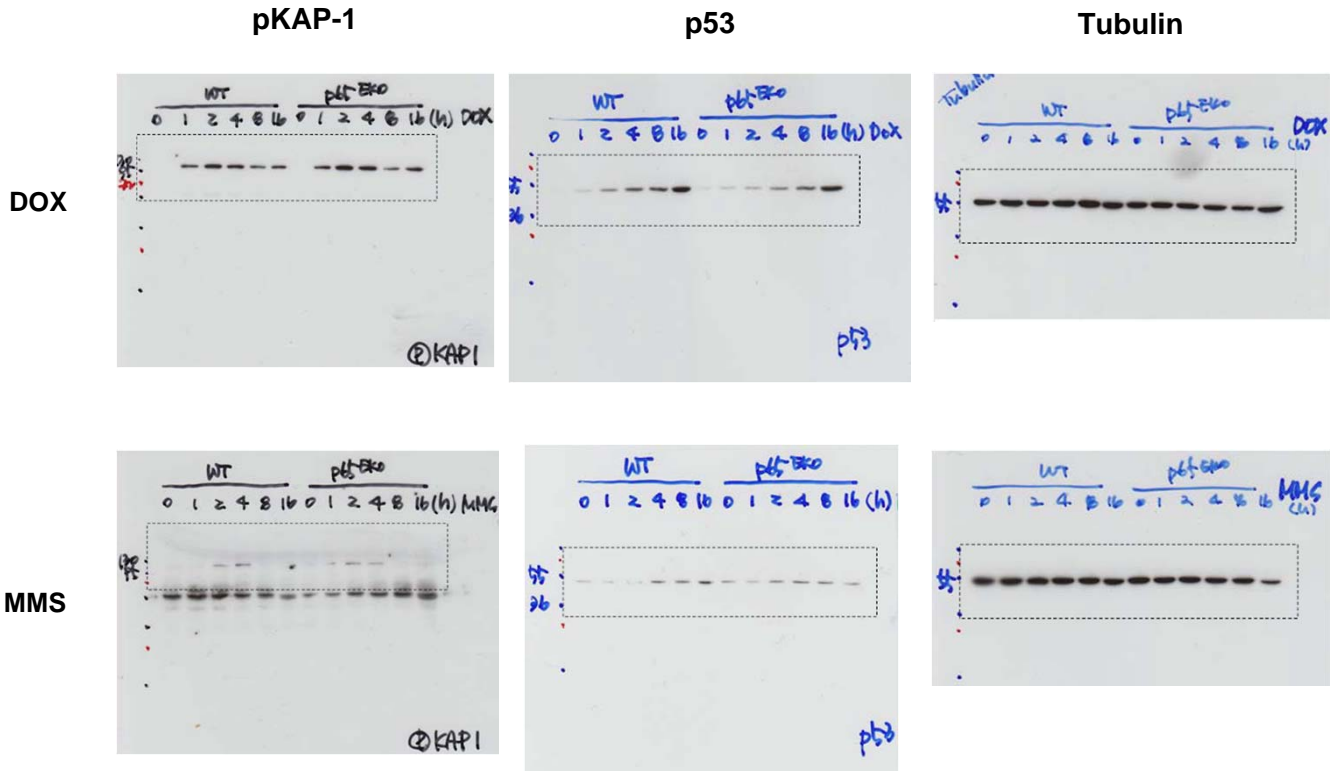

Supplement: Supplementary file 7 — Source Data for Figure 3 B [file emmm0006-0970-SD7.pdf]
