# Supplementary material for: Epidermal p65/NF-κB signalling is essential for skin carcinogenesis
Source: EMBO Mol Med. 2014 Jun 21;6(7):970–83. doi: 10.15252/emmm.201303541 (PMC4119358; doi:10.15252/emmm.201303541)
Supplement: Supplementary file 5 — Review Process File [file emmm0006-0970-SD5.pdf]

Epidermal p65/NF- B signalling is essential for skin carcinogenesis

Chun Kim and Manolis Pasparakis

Corresponding author: Manolis Pasparakis, University of Cologne

Review timeline:

Submission date:	02 October 2013
Editorial Decision:	06 November 2013
Revision received:	08 April 2014
Editorial Decision:	13 May 2014
Revision received:	16 May 2014
Editorial Decision:	19 May 2014
Revision received:	21 May 2014
Accepted:	22 May 2014

Transaction Report:

(Note: With the exception of the correction of typographical or spelling errors that could be a source of ambiguity, letters and reports are not edited. The original formatting of letters and referee reports may not be reflected in this compilation.)

Editor: Roberto Buccione

1st Editorial Decision 06 November 2013

We have now heard back from the three Reviewers whom we asked to evaluate your manuscript.

You will see that while the three Reviewers generally supportive of your work, they do raise a number of concerns that prevent us from considering publication at this time.

An issue raised by all three Reviewers is that the main contention that NF-kB is oncogene-like rather than a tumour suppressor in keratinocytes is considered to be insufficiently supported. I do ask you to respond carefully to the specific criticisms with further experimentation where necessary. For example by providing appropriate quantifications including Langerhans cell numbers in the p65EKO mice and gene expression studies where required.

Reviewers 1 and 2 are also concerned that the data conflict with a previous report and feel that you bear the responsibility (the "onus") to resolve the potential controversy stemming from your findings. I do not necessarily agree and while in depth discussion of the discrepancies is useful, the goal is ultimately to ensure your manuscript is of the quality and conclusiveness we expect for EMBO Molecular Medicine.

Considered all the above, while publication of the paper cannot be considered at this stage, we would be prepared to consider a revised submission, with the understanding that the Reviewers' concerns must be addressed as mentioned above, with additional experimental data where appropriate and that acceptance of the manuscript will entail a second round of review.

Please note that it is EMBO Molecular Medicine policy to allow a single round of revision only and

that, therefore, acceptance or rejection of the manuscript will depend on the completeness of your responses included in the next, final version of the manuscript.

As you know, EMBO Molecular Medicine has a "scooping protection" policy, whereby similar findings that are published by others during review or revision are not a criterion for rejection. However, I do ask you to get in touch with us after three months if you have not completed your revision, to update us on the status. Please also contact us as soon as possible if similar work is published elsewhere.

I look forward to seeing a revised form of your manuscript as soon as possible.

***** Reviewer's comments *****

Referee #1 (Remarks):

In this manuscript, Kim and Pasparakis have challenged the existing paradigm that NF- B transcription factor functions as a tumor suppressor in keratinocytes. Using a very well genetically defined model of ablating p65/RelA in the murine keratinocytes (p65EKO mice) they demonstrated that this protein is required for successful papilloma formation in the DMBA-TPA model of skin carcinogenesis. Inability of damaged cells to survive the DMBA assault along with poor inflammatory effects of TPA-mediated tumor promotion in p65EKO mice were proposed as the mechanisms underlying inefficient carcinogenesis in these animals. While this study has a number of exciting data (for example, role of keratinocyte-specific NF- B in TPA-induced inflammation), its publication appears premature.
General comments (in no particular order)
1. Authors have challenged the existing paradigm. They have discussed the potential reasons for the dichotomy between previously published data and their results at some length. However, while employing a "cleaner" genetic system, the challenge must be supported by a rigorous investigation rather than speculations. The onus is on the authors of this study to determine how varying the conditions (e.g. utilizing DMBA/TPA model in the I B SR transgenic mice or ablating p65 in RAS/ I B SR transgenics) will assert the role of NF- B as an oncogene or tumor suppressor in skin carcinogenesis.
2. The study is fairly descriptive and its conclusions on many occasions are based on correlative data. Some of these data are overinterpreted (see specific comments below).
3. The translational potential value of this study is unclear. Skin hyperplasia and benign papillomas observed in described experiments have not progressed to squamous cell carcinomas that may represent a clinically important problem. Furthermore, this study neither links the unveiled mechanisms and importance of p65 expression to human malignancies nor contains a proof of principle for a novel pharmacological approach to treating squamous cell carcinomas.
Specific comments:
1. Figure 1A should also have data on p65EHT animals.
2. Data shown in Figures 2C, 3C, 4A and 6B will benefit from statistical analysis
3. On page 5, authors describe correlative data shown in Figure 2 and conclude that "our results suggest that epidermal p65 deficiency protects mice from DMBA/TPA-induced skin tumorigenesis, at least in part, by sensitizing keratinocytes to DNA-damage induced death". In fact, there is a number of alternative possibilities - for example, the role of p65 in the repair of subtle DNA alterations such as RAS mutations post DMBA treatment.
4. On page 6, authors write: "Since NF- B has been proposed to antagonize the p53 pathway...". Available literature suggests that additional mechanisms should be considered. Ryan et al (Nature, 2000) proposed that NF- B activities are instrumental in p53-mediated cell death. Given that, a broader panel of pro-apoptotic p53 targets should be examined to supplement the results on Bbc3 shown in Figure 3C.
5. Data shown in Figure 4B demonstrating the differences between IAP2 responses to DOX versus MMS are not described or discussed in the text. These data are not entirely consistent with interpretation. It appears that p65-null keratinocytes still induce IAP2 by 24h after DOX yet lose it by 8h of MMS treatment. Regrettably, functional significance of these effects are not investigated for both DNA damaging agents.
6. Data in Figure 4C will be conclusive (and will support the interpretation in the text) only if authors manage to restore cellular IAP2 levels to that of p65EHT cells. Otherwise, protective effect

of forced expression of a known anti-apoptotic protein is rather uninformative.

Referee #2 (Comments on Novelty/Model System):

The relevance of these finding for human SCC development might be discussed in more detail.

Referee #2 (Remarks):

The paper by Kim and Pasparakis is focussing on the role of NF B in skin carcinogenesis using transgenic C57BL6 mice with a keratinocyte- specific deletion in p65. These mice were refractory to papilloma development when using the 2 stage model of skin carcinogenesis (DMBA/TPA) due to: i) increased apoptosis after DMBA, probably preventing tumour initiation, and ii) suppressing the skin hyperproliferation and the expression of proinflamatory cytokines and chemokines in keratinocytes following TPA treatment. These data are of high quality, original and of great interest for a broad readership of EMBO Molecular Medicine focussing on signal transduction, inflammation and cancer. However, there are a serious number of major points that need to be addressed by the authors.

1. A major concern comes from the fact that other groups have published totally opposing results: showing tumor suppressive functions of the NF B in epidermal keratinocytes (in contrast to other epithelial cells) as well as a marked hyperplasia in skin grafts derived from p65-/- embryos.
In this reviewer's opinion, the authors have to explained further the differences observed!

2. p65 epidermal KO (p65EKO) were resistant to tumour formation compared to heterozygous (p65EHT) or wt littermates. They present tumour incidence/numbers tumours per mouse histopathological analysis of DMBA-TPA treated skin at intermediate time points and at the final tumor stage are missing. What about the two only papillomas developed in the p65EKO group (n=21); are similar to those, which developed in the control group?
3. To confirm similar metabolism of DMBA and activation of DDR in p65EKO mice (Fig 2A) the authors performed a single application with 400 nmol DMBA followed by IHC staining for gammaH2AX. Did they obtain similar findings with 100 nmol, corresponding to the concentration of the chemically induced carcinogenesis protocol? It will be helpful to investigate by IHC staining whether keratinocyte-specific p65 deletion has an impact on the amount of Langerhans cells that are present within the epidermis?
2. As already mentioned by the authors p53 immunostaining was detectable in keratinocytes of the epidermis from untreated p65EKO and K14-Cre, but not in p65FL mice (Suppl Fig. 2). However, no TUNEL-positive keratinocytes were detected in these mice without DMBA treatment (Fig. 2B). The authors should exclude the possibility that the presence of the Cre transgene induces spontaneous keratinocyte senescense in vivo as well as in vitro, which in combination with ablation of p65 may affects tumorigenesis.
3. In the past, it was shown that deletion of Nemo or IKK2, accompanied by reduced levels of p65 activity, resulted in impaired proliferation of primary keratinocytes in culture (Nenci et al, 2006; Pasparakis et al, 2002). Is this also true for primary keratinocytes in culture derived from p65EKO mice?
4. The authors were not able to detect significant down-regulation of distinct NF-kB dependent survival genes in DOX- or MMS- treated p65-deficient keratinocytes compared to similarly treated wild type cells (Fig 4A and Fig S3 of Supporting Information). Is this also true for DMBA-treated back skin of p65FL and p65EKO mice?

5. The authors transfected a Birc3-expressing construct into p65-deficient keratinocytes and monitored their cell viability after DOX treatment (Fig 4C). However, they provide no information on the transfection efficiency or show comparable amounts cIAP2 protein levels in p65FL and transfected p65EKO cells by Western blot analysis. Moreover, aberrant cIAP2 protein levels in untreated and DMBA-treated epidermal keratinocytes of p65EKO as compared to p65FL animals is urgently needed to further support their conclusion. Does depletion of Birc3 in wt keratinocytes promote apoptosis to a similar level of p65 deficient cells after DOX treatment? Why did the authors use different types of DNA damage agent? Please explain. The technical quality of detection of cIAP2 by WB needs improvement.

6. Single TPA application induces acute inflammation and does not resemble conditions of chronic inflammation that are required for tumour promotion in DMBA/TPA-induced skin carcinogenesis. The authors have to investigate skin hyperplasia and keratinocyte proliferation as well as the quality and quantity of infiltrating stromal immune cells following repeated TPA treatment with or without DMBA pretreatment. Furthermore, FACS analysis should be done for better classification and quantification of infiltrating immune cell sub-populations and to support IHC staining data.
7. Up-regulation of TNFalpha and Cxcl2 in mouse back skin is shown 48 hours upon TPA-induction, but is not present at 24 hours. In contrast, primary keratinocytes exhibit a much faster kinetic of chemokine induction. Since, both cytokines are also produced by activated macrophages and granulocytes, altered levels between p65FL and p65EKO skin could simple be a consequence of different numbers of inflammatory cells and not the initial cause for the observed phenotype. Consequently, the authors have to confirm reduced cytokine expression by: (i) expression analysis with RNA or protein derived from the epidermis or (ii) in situ hybridization or IHC analysis of skin sections. Why don't they show skin sections of 1x DMBA treated and once TPA treated like it's done in other similar studies (Fig 2 Johansen C, carcinogenesis 2009)?
8. A main conclusion of the manuscript is that inhibition of NF-kB prevents tumour initiation by facilitating the clearance of cells bearing damaged DNA. However, does the epidermis of DMBA-initiated p65FL mice exhibit an increased number of keratinocytes with Ras mutations and more mutations in the genome as compared to p65EKO mice?
9. This reviewer likes to see histological sections of the papillomas at week 9 and 21, corroboration of fig 4 in the skin of the animals, demonstration of Birc3 overexpression in cell culture, whereas fig 3B and C could perfectly go to supplemental figures.

Referee #3 (Remarks):

The study by Kim and Pasparakis investigates the role of in keratinocyte p65 in the development of skin tumors after DMBA/TPA treatment. The authors found that keratinocyte-specific p65 knock-out inhibits tumorigenesis and inflammatory skin hyperplasia. This is in accordance with a role of NF-kappaB in promoting the survival of mutagenized cells and in enhancing tumor progression by establishing a pro-inflammatory environment. The study is generally well performed and the manuscript is well organized. However, the interpretation of some experiments should be supported by more quantitative data. The paper could be improved by addressing following concerns:

1. The selection of analyzed NF-kappaB-inducible anti-apoptotic genes is not clear. There are several other genes that could be tested, Tnfaip3 and Bcl2a1a for example. Both are highly NF-kappaB inducible and anti-apoptotic. The fact that cIAP2 rescues cell survival after DOX treatment cannot be used as an indication that cIAP2 has a prominent role in vivo. Furthermore, in Fig 4C, tubulin expression seems to be lower in p65EKO mice. How does that impact the interpretation of the blots? Quantification and normalization of the signal would be helpful.

2. One major questions remains whether the findings are model specific or could be laso observed in skin carcinogenesis models where the inflammatory component is not so prominent as in the DMBA/TPA model.

3. Fig 5. Skin hyperplasia should be measured and presented as average epidermal thickness or number of nucleated interfollicular cell layers in all experimental groups.

4. The strain and gender of mice used in the study are not indicated. Authors should also disclose randomization and blinding protocols.

1st Revision - authors' response 08 April 2014

Point by point response

Reviewer 1

Remarks:

In this manuscript, Kim and Pasparakis have challenged the existing paradigm that NF-κB transcription factor functions as a tumor suppressor in keratinocytes. Using a very well genetically defined model of ablating p65/RelA in the murine keratinocytes (p65EKO mice) they demonstrated that this protein is required for successful papilloma formation in the DMBA-TPA model of skin carcinogenesis. Inability of damaged cells to survive the DMBA assault along with poor inflammatory effects of TPA-mediated tumor promotion in p65EKO mice were proposed as the mechanisms underlying inefficient carcinogenesis in these animals. While this study has a number of exciting data (for example, role of keratinocyte-specific NF-κB in TPA-induced inflammation), its publication appears premature.

General comments (in no particular order)

1. Authors have challenged the existing paradigm. They have discussed the potential reasons for the dichotomy between previously published data and their results at some length. However, while employing a "cleaner" genetic system, the challenge must be supported by a rigorous investigation rather than speculations. The onus is on the authors of this study to determine how varying the conditions (e.g. utilizing DMBA/TPA model in the IκBαSR transgenic mice or ablating p65 in RAS/IκBαSR transgenics) will assert the role of NF-κB as an oncogene or tumor suppressor in skin carcinogenesis.

With all respect to the reviewer's opinion, we disagree with this comment. The reviewer writes, "***the challenge must be supported by a rigorous investigation rather than speculations".*** In fact, we do not see our manuscript as a challenge to the already published studies. We do not claim that the previous studies are wrong and our studies are right. Instead, we present our data and we discuss the differences in the two models used that could explain the different phenotypes observed. Therefore, we do not see our paper as a "challenge" to prove the previous studies wrong, but rather as a study presenting an alternative view of the role of epithelial NF-κB signalling in skin tumourigenesis. Therefore, the question is whether our data is sufficient to demonstrate that in our mouse model and in the carcinogenesis protocol we used, p65/NF-κB signalling has a tumour-suppressing or tumour-promoting function. A clear answer to this question is given by our *in vivo* results, which demonstrate that p65 knockout in keratinocytes prevented tumour development induced by DMBA/TPA. The reviewers do not question this result. In fact this reviewer states above: *"Using a **very well genetically defined model** of ablating p65/RelA in the murine keratinocytes (p65EKO mice) **they demonstrated that this protein is required for successful papilloma formation in the DMBA-TPA model of skin carcinogenesis**."*

We therefore disagree with the reviewer that the “onus” is on us to clarify why our results are different from the previously published studies. These are studies performed in different mice and different models of tumourigenesis and we believe our paper will provide an alternative view of the role of NF-κB in skin cancer and will sparkle new discussions and new studies in this direction.

2. The study is fairly descriptive and its conclusions on many occasions are based on correlative data. Some of these data are overinterpreted (see specific comments below).

The main conclusion of our manuscript is that epidermal p65 is essential for DMBA/TPA-induced tumour development. We believe that we provide *in vivo* data in an appropriate genetic mouse model that clearly support this conclusion. In fact, this part of our study is not questioned by any of the reviewers. Our *in vivo* and in vitro experiments on the mechanisms by which p65 knockout prevents skin tumour development are to some extent correlative as they are based in part on *ex vivo* experiments performed on cells cultured *in vitro*. However, we have performed these experiments in primary keratinocytes, which are the closest *in vitro* system to the epidermis, and not in cancer cell lines that are often used in such experiments. Given that our *in vitro* results correlate well with our *in vivo* findings we believe it is legitimate to use these results to support our conclusions. We should point out here that it is a well-accepted practice in experimental research on animal models to perform mechanistic studies in primary cells cultured ex vivo. Such ex vivo studies are often necessary when it is not possible to perform controlled stimulations in the context of the whole tissue *in vivo*. In addition, ethical guidelines and the 3R principle strongly encourages the use of *in vitro* systems replacing *in vivo* experiments in order to reduce unnecessary animal suffering. Therefore, while we agree with the reviewer that the *in vitro* data are to some extent correlative, we disagree that our data are overinterpreted as we believe they are discussed in a balanced manner.

3. The translational potential value of this study is unclear. Skin hyperplasia and benign papillomas observed in described experiments have not progressed to squamous cell carcinomas that may represent a clinically important problem. Furthermore, this study neither links the unveiled mechanisms and importance of p65 expression to human malignancies nor contains a proof of principle for a novel pharmacological approach to treating squamous cell carcinomas.

The DMBA/TPA model induces papillomas that do not progress to carcinomas in the C57BL/6 genetic background, as is the case in other more tumour prone strains such as FVB/N. Therefore, our studies described here are necessarily restricted to tumour initiation and the formation of papillomas, and cannot address progression to carcinomas. With regards to its translational potential, as every mouse model study, our work presented here suggests but does not provide proof that a similar mechanism also applies in human skin cancer. We used a well-established and universally accepted model of two-stage skin tumourigenesis to address a specific question in a clearly defined genetic mouse model. Addressing the clinical significance of our findings for human skin cancer was not our intention and although

this is certainly a very important topic it is, to our opinion, outside the scope of this study.

Specific comments:

1. Figure 1A should also have data on p65EHT animals.

We now included epidermis from $p65^{EHT}$ animals in new Figure 1A.

2. Data shown in Figures 2C, 3C, 4A and 6B will benefit from statistical analysis

We include statistical analysis in the revised manuscript.

*3. On page 5, authors describe correlative data shown in Figure 2 and conclude that "our results suggest that epidermal p65 deficiency protects mice from DMBA/TPA-induced skin tumorigenesis, **at least in part**, by sensitizing keratinocytes to DNA-damage induced death". In fact, there is a number of alternative possibilities - for example, the role of p65 in the repair of subtle DNA alterations such as RAS mutations post DMBA treatment.*

We respectfully disagree with the reviewer's description of our data shown in Figure 2 as correlative. Our conclusion is based on our findings that treatment with genotoxic drugs induces the death of considerably more keratinocytes in the epidermis of $p65^{EKO}$ mice *in vivo* (DMBA experiment, Figure 2B and Supplementary Figure 2) and *in vitro* (DOX and MMS experiments, Figure 2C). We believe these results clearly demonstrate that p65 deficiency sensitizes keratinocytes to DNA damage-induced death. Based on the well-accepted role of DNA damage-induced cell death as a mechanism preventing carcinogen-induced tumour development, we therefore suggest that sensitization of keratinocytes to DNA damage induced death is **at least one of the mechanisms** by which keratinocyte p65 deficiency protects mice from DMBA/TPA-induced skin tumourigenesis. We did not claim this is the only mechanism. In fact in the vast majority of *in vivo* studies in genetic mouse models there is more than one mechanism by which a knockout affects a tissue response *in vivo*. Of course we cannot exclude that other mechanisms also contribute to the suppression of tumourigenesis in the epidermis of $p65^{EKO}$ mice, but our results clearly show that sensitization to DNA damage-induced death contributes to the resistance of $p65^{EKO}$ mice to DMBA/TPA-induced skin tumours.

4. On page 6, authors write: "Since NF-κB has been proposed to antagonize the p53 pathway...". Available literature suggests that additional mechanisms should be considered. Ryan et al (Nature, 2000) proposed that NF-κB activities are instrumental in p53-mediated cell death. Given that, a broader panel of pro-apoptotic p53 targets should be examined to supplement the results on Bbc3 shown in Figure 3C.

The relationship between NF-κB and p53 during DNA damage has been addressed in various studies that provided often conflicting results and therefore remains

controversial. On the one hand, some studies including the work of Ryan at al cited by the reviewer (Ryan *et al*, 2000) claim that p53 is a prerequisite for NF-κB activation and that p53-mediated NF-κB activation plays a merely apoptotic role. On the other hand, other studies such as the work by Tergaonkar et al (Tergaonkar *et al*, 2002) convincingly demonstrated that genotoxic stress-induced p53 and NF-κB activation represent two independent parallel pathways that may interact with each other's action through distinct mechanisms. Being aware of these conflicting reports, we wanted to explore a potential impact of p65 deficiency on p53 signalling. Our analysis of p53 activation was not biased by any of these earlier studies. Our results showed that the lack of NF-κB/p65 activity had no measurable impact on genotoxin-induced p53 stabilization *in vivo* and *in vitro*. Also, as shown in Fig. 3C, the expression of the major p53 dependent apoptotic genes encoding Bax, Puma and p21 was not upregulated in p65-deficient keratinocytes. Based on these results we concluded that p65 deficiency did not result in altered DNA damage-induced activation of p53. Nevertheless, following the reviewer`s suggestion, we analyzed the expression of more p53 dependent proapoptotic genes including *Bcl2L11, Bid,* and *Bak1*, which encode Bim, Bid and Bak, respectively. These data are now included in the revised manuscript in Figure S4 and confirm that the absence of p65 in keratinocytes did not alter significantly the expression of these genes, although we did detect a trend towards decreased expression of *Bcl2l11* in $p65^{EKO}$ keratinocytes.

5. *Data shown in Figure 4B demonstrating the differences between IAP2 responses to DOX versus MMS are not described or discussed in the text. These data are not entirely consistent with interpretation. It appears that p65-null keratinocytes still induce IAP2 by 24h after DOX yet lose it by 8h of MMS treatment. Regrettably, functional significance of these effects are not investigated for both DNA damaging agents.*

We appreciate the insightful comments of the reviewer regarding cIAP2. During repeating our experiments we realized there were inconsistencies with the bands detected by the anti-cIAP2 antibodies we used. We sought advise from expert labs working with cIAP2 proteins and learned that there is currently no antibody that reliably detects mouse cIAP2 by immunoblotting. This information prompted us to rigorously evaluate the specificity of the anti-cIAP2 antibody we used. We therefore performed a control experiment utilizing cIAP2 KO MEFs and LBW242, a smac mimetic compound (SM) that induces degradation cIAP1 and cIAP2.

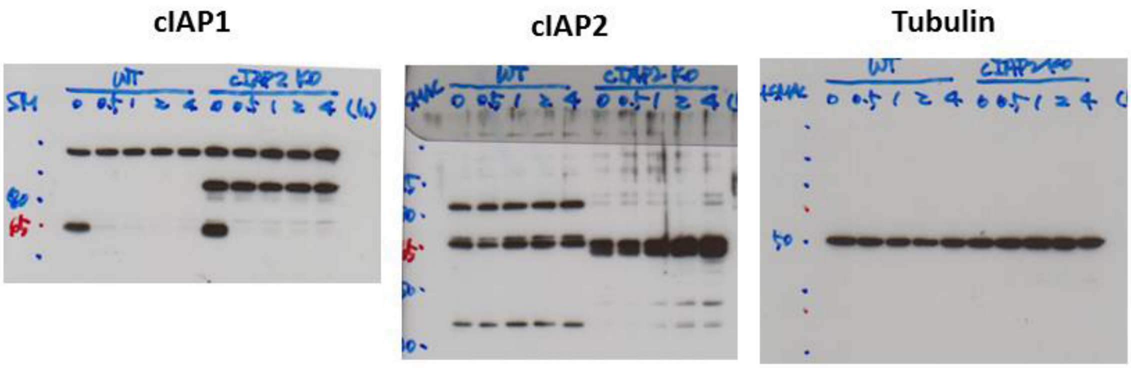

As shown in our previous manuscript with keratinocytes, the anti-cIAP2 antibody detected a band at the expected size (slightly above ~65kDa) in WT MEFs. However, the very same band also appeared in cIAP2 KO MEFs. Although we also see that the antibody detects another slightly larger protein only in WT MEFs, we believe this is not cIAP2 because it is not affected by SM treatment, which should induce the degradation of both cIAP1 and cIAP2. As shown in the anti-cIAP1 blot, SM efficiently induced the degradation of cIAP1 (~65kDa) in both WT and cIAP2 MEFs within 30 min, indicating that the SM treatment was successful. These experiments showed that we cannot rely on the anti-cIAP2 antibody for the detection of cIAP2 protein levels in mouse cells and tissues. We therefore decided to remove Figure 4B from the manuscript and rely solely on qPCR analysis of mRNA levels of the Birc3 gene encoding cIAP2 in our experiments. As shown in Fig. 4A, p65 knockout keratinocytes showed impaired Birc3 expression in response to both DOX and MMS, therefore there is not discrepancy in the data obtained using these two different DNA damage-inducing agents.

6. *Data in Figure 4C will be conclusive (and will support the interpretation in the text) only if authors manage to restore cellular IAP2 levels to that of p65EHT cells. Otherwise, protective effect of forced expression of a known anti-apoptotic protein is rather uninformative.*

Figure 4C is now relocated to Figure4B. Given that these experiments are performed in primary keratinocytes, it is not possible to prepare stable clones and select those with expression levels equivalent to $p65^{EKO}$. We agree with the reviewer that the data based on overexpressing cIAP2 should be discussed with caution and this is what we tried to do in our paper. However, the pro-survival role of cIAP2 is well established, also in protecting cells from DNA damage induced death. We therefore believe that the reduced expression of cIAP2 in the p65-deficient cells together with the data showing that (over)expression of cIAP2 protects p65-deficient cells from DNA damage-induced death support our suggestion that reduced cIAP2 levels are likely to contribute to the sensitization of p65 knockout keratinocytes to DNA damage-induced death.

Referee #2 for EMM-2013-03541:

Remarks:

The paper by Kim and Pasparakis is focussing on the role of NF-κB in skin carcinogenesis using transgenic C57BL6 mice with a keratinocyte- specific deletion in p65. These mice were refractory to papilloma development when using the 2 stage model of skin carcinogenesis (DMBA/TPA) due to: i) increased apoptosis after DMBA, probably preventing tumour initiation, and ii) suppressing the skin hyperproliferation and the expression of proinflamatory cytokines and chemokines in keratinocytes following TPA treatment. These data are of high quality, original and of great interest for a broad readership of EMBO Molecular Medicine focussing on

signal transduction, inflammation and cancer. However, there are a serious number of major points that need to be addressed by the authors.

1. A major concern comes from the fact that other groups have published totally opposing results: showing tumor suppressive functions of the NF-κB in epidermal keratinocytes (in contrast to other epithelial cells) as well as a marked hyperplasia in skin grafts derived from p65-/- embryos. In this reviewer's opinion, the authors have to explained further the differences observed!

The reviewer raises two different issues here. The first relates to the study by Khavari and colleagues suggesting that NF-κB signalling in keratinocytes has tumour suppressive functions (Dajee *et al*, 2003), and the second relates to another study from the same group showing that skin from p65-deficient embryos develops hyperplasia when grafted in adult hosts (Zhang *et al*, 2004). Starting from the latter study, indeed our results showing the epidermal keratinocyte-restricted ablation of p65 does not cause spontaneous epidermal hyperplasia contradicts the findings of Zhang et al and suggests that under normal conditions p65 signalling in keratinocytes is dispensable for normal epidermal proliferation, differentiation and homeostasis. We would like to point out that another independent study of mice with epidermal keratinocyte restricted p65 knockout performed in a different lab using another p65 floxed mouse and another K5-Cre transgenic mouse found the same results as we did, namely that mice with keratinocyte restricted ablation of p65 have normal skin and do not show epidermal hyperplasia (Rebholz *et al*, 2007). Therefore, two independent studies using Cre-mediated conditional ablation of p65 in keratinocytes failed to detect a role of p65 in controlling normal epidermal proliferation or differentiation. It is unclear to us why these experiments reached a different result compared to the study of Zhang et al (Zhang *et al*, 2004). We can only speculate that the nature of the experimental setup, which is very different in these studies may be responsible for the different results. However, irrespective of the reason behind this apparent contradiction, we believe that our studies backed by those of Rebholz et al (Rebholz *et al*, 2007) convincingly demonstrate that p65 is dispensable for the regulation of epidermal development and homeostasis under normal conditions.

With regards to the cancer studies, as we discussed above in our response to reviewer 1, we do not claim that our studies are correct and the studies reported by Dajee et al are wrong. Instead, we present our data and we discuss the differences in the two models used that could explain the different phenotypes observed. Therefore, we do not see our paper as a “challenge” to prove the previous studies wrong, but rather as a study presenting an alternative view of the role of epithelial NF-κB signalling in skin tumourigenesis. Therefore, the question is whether our data is sufficient to demonstrate that in our mouse model and in the carcinogenesis protocol we used, p65/NF-κB signalling has a tumour-suppressing or tumour-promoting function. A clear answer to this question is given by our *in vivo* results, which demonstrate that p65 knockout in keratinocytes prevented tumour development induced by DMBA/TPA. The reviewers do not question this result, on the contrary they agree that our finds show that mice with p65 ablation in keratinocytes are protected from DMBA/TPA-induced skin tumour development. We believe it is unfair to put the load on our studies to explain why our results are

different from the previously published work by Dajee et al. These studies are performed in different mice and different models of tumourigenesis and we believe our work will provide an alternative view of the role of NF-κB in skin cancer and will sparkle new discussions and new studies in this direction.

2. p65 epidermal KO (p65EKO) were resistant to tumour formation compared to heterozygous (p65EHT) or wt littermates. They present tumour incidence/numbers tumours per mouse histopathological analysis of DMBA-TPA treated skin at intermediate time points and at the final tumor stage are missing. What about the two only papillomas developed in the p65EKO group (n=21); are similar to those, which developed in the control group?

As stated in the manuscript, only 2 out of 21 p65EKO mice developed a small single papilloma. These papillomas were small and underdeveloped compared to the papillomas found in the control p65FL animals. Below we include representative macroscopic and histological images of these papillomas. Since the histopathology of DMBA/TPA induced papillomas is well characterised in numerous previous publications, we did not consider important to present these results in the main manuscript. Also, we do not believe that the small single papillomas that developed in two p65^{EKO} mice can represent the phenotype of this group. For this reason we include this figure here for the reviewers. However, if the reviewers consider important to present these results in the manuscript we would be happy to include this figure.

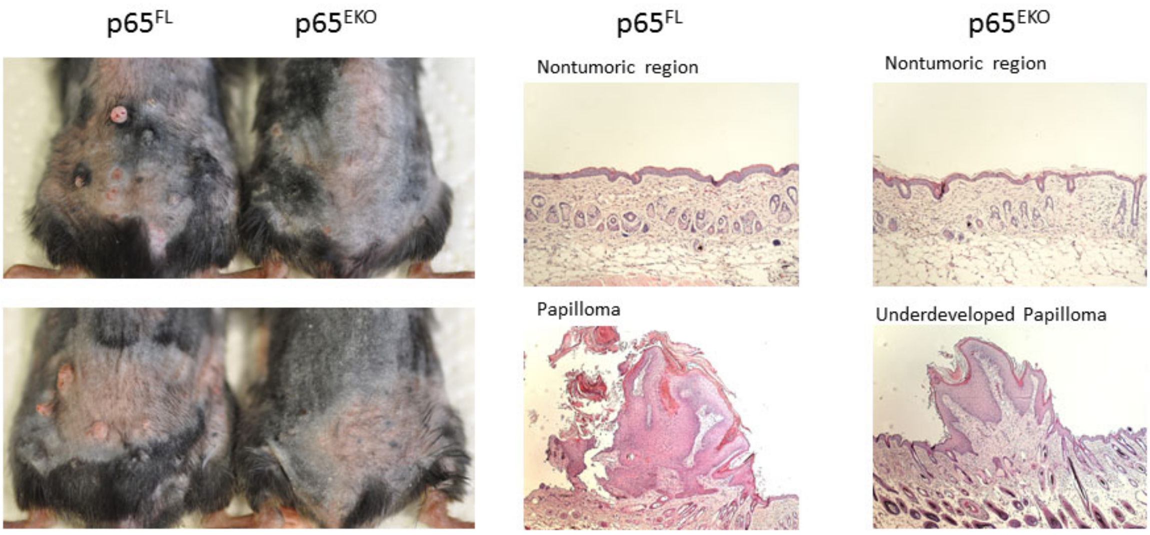

3. To confirm similar metabolism of DMBA and activation of DDR in p65EKO mice (Fig 2A) the authors performed a single application with 400 nmol DMBA followed by IHC staining for gammaH2AX. Did they obtain similar findings with 100 nmol, corresponding to the concentration of the chemically induced carcinogenesis protocol? It will be helpful to investigate by IHC staining whether keratinocyte-specific p65 deletion has an impact on the amount of Langerhans cells that are present within the epidermis?

The purpose of these experiments was to rule out the possibility that different metabolism of DMBA in the skin of $p65^{EKO}$ mice contributes to the phenotype. Our analysis of gamma-H2AX staining in the epidermis of these mice showed similar DNA damage response in keratinocytes of WT and $p65^{EKO}$ mice, ruling out the possibility that the resistance of $p65^{EKO}$ mice to tumour development could be due to impaired conversion of DMBA to its active metabolite. This result suggests that the number of LCs and their capacity to metabolize DMBA is similar in the WT and KO groups. Nevertheless, in response to the reviewer's comment we assessed LC numbers in the epidermis of WT and KO mice and found that epidermal p65 deficiency did not alter the number of LCs. These results are now included in Figure S1 of the revised manuscript.

With regards to the dose of DMBA used, we performed the gamma-H2AX analysis using the 400 nmol dose but have compared the 400 nmol and 100 nmol doses to assess the induction of DNA damage-induced death (Figure 2B and Figure S2). Both doses of DMBA gave a similar result (more keratinocyte death in $p65^{EKO}$ mice), with the difference that the overall number of dead cells was lower in the 100 nmol dose.

2. As already mentioned by the authors p53 immunostaining was detectable in keratinocytes of the epidermis from untreated p65EKO and K14-Cre, but not in p65FL mice (Suppl Fig. 2). However, no TUNEL-positive keratinocytes were detected in these mice without DMBA treatment (Fig. 2B). The authors should exclude the possibility that the presence of the Cre transgene induces spontaneous keratinocyte senescense in vivo as well as in vitro, which in combination with ablation of p65 may affects tumorigenesis.

Our results showing that the $p65^{EHT}$ mice, which express the Cre transgene and have heterozygous p65 deletion in the epidermis, developed tumours similarly to the $p65^{FL/FL}$ mice that do not express the Cre transgene demonstrate that the Cre transgene by itself or in combination with heterozygous p65 knockout does not affect DMBA/TPA induced skin tumourigenesis. This is the best control that we can use to exclude a potential Cre effect in these studies. The reviewer raises the question whether the presence of the Cre transgene in combination with the loss of both p65 alleles might affect tumourigenesis. Although at this stage there is no evidence suggesting that Cre expression induces senescence in the keratinocytes of K14Cre mice (we have analysed a very large number of epidermis specific knockouts using this transgene and also have kept K14Cre mice aging for more than a year and have not seen any effects induced solely by the Cre expression), we cannot exclude the theoretical possibility that Cre expression in combination with full loss of p65 might have an additional effect. In fact this is a potential theoretical concern for any study using Cre/loxP mediated cell specific knockout mouse models, which probably included a few thousand published studies by now. However, given the nature of the model requiring Cre expression to delete the floxed genes, it is not possible technically to address this possibility.

3. In the past, it was shown that deletion of Nemo or IKK2, accompanied by reduced levels of p65 activity, resulted in impaired proliferation of primary keratinocytes in

culture (Nenci et al, 2006; Pasparakis et al, 2002). Is this also true for primary keratinocytes in culture derived from p65EKO mice?

We have not performed a comprehensive analysis of the proliferation of p65 knockout keratinocytes *in vitro* since we did not see any difference in epidermal keratinocyte proliferation and differentiation *in vivo*, which is a more important and relevant results for our studies presented here. Our impression is that the p65-deficient keratinocytes indeed grow a little slower than control cells but we believe that a comprehensive analysis of the proliferation of p65 knockout keratinocytes *in vitro* is outside the scope of the current manuscript.

4. The authors were not able to detect significant down-regulation of distinct NF-kB dependent survival genes in DOX- or MMS- treated p65-deficient keratinocytes compared to similarly treated wild type cells (Fig 4A and Fig S3 of Supporting Information). Is this also true for DMBA-treated back skin of p65FL and p65EKO mice?

It is technically not feasible to study the effect of DMBA treatment in epidermal keratinocyte gene expression *in vivo*. This is because it is not possible to prepare clean epidermal sheaths from the back skin of adult mice. Even if we could try to prepare small pieces of clean epidermis, which as stated above is technically really very challenging in adult mice, this would include extensive incubation of the skin in enzymatic solutions that is likely to have unpredictable effects in the gene expression profile of the cells. Also, gene expression analysis of the whole skin would not be meaningful as it would be impossible to dissect the contribution of epidermal versus non-epidermal cells. For this reason, we resorted to using primary epidermal keratinocytes culture *in vitro*, which is to our opinion the best system to directly address the role of p65 in DNA damage-induced expression of prosurvival genes.

5. The authors transfected a Birc3-expressing construct into p65-deficient keratinocytes and monitored their cell viability after DOX treatment (Fig 4C). However, they provide no information on the transfection efficiency or show comparable amounts cIAP2 protein levels in p65FL and transfected p65EKO cells by Western blot analysis. Moreover, aberrant cIAP2 protein levels in untreated and DMBA-treated epidermal keratinocytes of p65EKO as compared to p65FL animals is urgently needed to further support their conclusion. Does depletion of Birc3 in wt keratinocytes promote apoptosis to a similar level of p65 deficient cells after DOX treatment? Why did the authors use different types of DNA damage agent? Please explain. The technical quality of detection of cIAP2 by WB needs improvement.

As explained above in our response to comment #5 of reviewer 1, after extensive and time consuming efforts to improve the cIAP2 immunoblotting we realized that there are no antibodies that can detect mouse cIAP2. We therefore regret that we are no able to assess cIAP2 protein levels in our mice and cells and therefore have to rely on mRNA levels of *Birc3*, the gene encoding cIAP2. We agree with the reviewer that the data based on overexpressing cIAP2 should be discussed with caution and this is what we tried to do in our paper. However, the pro-survival role of cIAP2 is well established, also in protecting cells from DNA damage induced death.

We therefore believe that the reduced expression of cIAP2 in the p65-deficient cells together with the data showing that (over)expression of cIAP2 protects p65-deficient cells from DNA damage-induced death support our suggestion that reduced cIAP2 levels are likely to contribute to the sensitization of p65 knockout keratinocytes to DNA damage-induced death. We used MMS and DOX as two widely used DNA damaging agents, since it is not possible to use DMBA *in vitro* as we described in the manuscript.

6. Single TPA application induces acute inflammation and does not resemble conditions of chronic inflammation that are required for tumour promotion in DMBA/TPA-induced skin carcinogenesis. The authors have to investigate skin hyperplasia and keratinocyte proliferation as well as the quality and quantity of infiltrating stromal immune cells following repeated TPA treatment with or without DMBA pretreatment. Furthermore, FACS analysis should be done for better classification and quantification of infiltrating immune cell sub-populations and to support IHC staining data.

Our *in vivo* results show clearly that $p65^{EKO}$ mice develop less hyperplasia and show reduced inflammatory cell infiltration in response to TPA treatment. Combined with our *in vitro* data showing that p65 deficiency very strongly impairs TPA-induced expression of inflammatory cytokines, we believe these results fully support our conclusion that epidermal p65 is required for the efficient induction of TPA-mediated tumour promoting inflammation. An extensive study of the inflammatory infiltrate as suggested by the reviewer might be potentially useful to finely define the detailed effect of p65 deficiency in the TPA induced inflammatory response. However, considering the time and costs associated with such a study and also ethical issues arising from having to expose large numbers of animals to TPA treatment for multiple time points which is clearly against the 3R principle we are obliged to comply to, we believe that such an extensive *in vivo* analysis of the inflammatory infiltrate is not justified.

7. Up-regulation of TNFalpha and Cxcl2 in mouse back skin is shown 48 hours upon TPA-induction, but is not present at 24 hours. In contrast, primary keratinocytes exhibit a much faster kinetic of chemokine induction. Since, both cytokines are also produced by activated macrophages and granulocytes, altered levels between p65FL and p65EKO skin could simple be a consequence of different numbers of inflammatory cells and not the initial cause for the observed phenotype. Consequently, the authors have to confirm reduced cytokine expression by: (i) expression analysis with RNA or protein derived from the epidermis or (ii) in situ hybridization or IHC analysis of skin sections. Why don't they show skin sections of 1x DMBA treated and once TPA treated like it's done in other similar studies (Fig 2 Johansen C, carcinogenesis 2009)?

The study cited by the reviewer (Johansen *et al*, 2009) shows in figure 2 results from the expression analysis of cytokines in punch biopsies from the skin of WT and KO mice treated with one application of DMBA followed by 4 applications of TPA. Punch biopsies encompass the entire skin and therefore cannot address epidermal specific

gene expression. For this specific study cited by the reviewer this was not important anyway since the knockout mice analysed were conventional (TNF-/- and MK2-/-) and it was not possible to dissect epidermal versus non epidermal cell contribution to the cytokine expression. In our case, we should stress that while indeed the expression of cytokines in full skin could be due to expression in epithelial and non epithelial cells, we study an epidermis-specific knockout animal. Performing the RT-PCR analysis of DMBA- or TPA-induced gene expression on keratinocytes isolated from DMBA or TPA animals is technically not feasible. As described in the methods section, our keratinocyte isolation method includes epidermis-dermis separation by enzymatic digestion of newborn mouse skin. Isolation of epidermal sheaths from adult mouse skin is technically challenging (only performed routinely in tail skin that is different from the back skin that is used as the target site in DMBA/TPA induced tumourigenesis) and nevertheless requires extensive enzymatic digestion steps that are likely to induce artifacts. Therefore, even if we could succeed in isolating keratinocytes from *in vivo* treated mice their gene expression profile would likely be affected by the procedures used to isolate the epidermal cells and would not accurately reflect the changes induced by the specific treatment.

However, we would like to point out that our approach to use primary epidermal keratinocytes to perform mechanistic studies to support our *in vivo* findings is widely accepted practice in experimental biology. The use of primary cells from genetically modified mice overcomes many of the artifacts inherent with the use of cell lines and overexpression systems that were used routinely in the past. We, and we believe a big part of the scientific community, are therefore convinced that using primary cell cultures for mechanistic studies complementing *in vivo* experiments is a valid approach to bring together the best of two worlds: *in vivo* studies are ideal to address the physiological significance of specific genes and pathways in complex disease related processes (e.g. tumour development) but the inaccessibility, complex cellular composition and architecture of tissues and organs make it very difficult, often impossible, to perform mechanistic studies in a time-resolved manner to address the cell-intrinsic functions of the studied genes in living animals; *in vitro* (or *ex vivo*) studies in primary cells is often the next best system to address mechanistic questions that are then related to the *in vivo* findings. We therefore believe that our approach to use *in vitro* studies on primary keratinocytes to address mechanistic questions relevant to our *in vivo* findings is valid and provides valuable information that could not be obtained *in vivo*.

8. A main conclusion of the manuscript is that inhibition of NF-kB prevents tumour initiation by facilitating the clearance of cells bearing damaged DNA. However, does the epidermis of DMBA-initiated p65FL mice exhibit an increased number of keratinocytes with Ras mutations and more mutations in the genome as compared to p65EKO mice?

Our conclusion is based on the very clear phenotype of the $p65^{EKO}$ mice, which demonstrates that p65 deficiency specifically in keratinocytes essentially completely prevents DMBA/TPA-induced tumour development, supported by our *in vivo* and in vitro studies showing that p65 knockout keratinocytes are more prone to undergo cell death in response to DNA damage and the well accepted tumour suppressing

function of DNA damage induced cell death. We believe that a quantitative analysis of the number of keratinocytes with Ras mutations (single cell sequencing?) or overall genomic mutations (Array cGH, exome sequencing?) is outside the scope of this manuscript.

9. This reviewer likes to see histological sections of the papillomas at week 9 and 21, corroboration of fig 4 in the skin of the animals, demonstration of Birc3 overexpression in cell culture, whereas fig 3B and C could perfectly go to supplemental figures.

Since the papillomas develop only in the WT animals, we do not see the added value of the analysis of papillomas at week 9 and 21 on the skin of wild type mice for our current study. This is a well-established model and the nature of papillomas induced in different stages in C57Bl/6 mice is also well characterized by previous studies. As discussed above, it is not technically possible to address DNA damage induced gene expression in keratinocytes from the back skin of adult mice treated with DMBA. Also, as discussed above, since there are no cIAP2 antibodies that detect mouse cIAP2 we regret that we cannot provide analysis of cIAP2 protein expression. However, we believe that our *in vivo* and *in vitro* experiments fully support our conclusion that reduced cIAP2 expression likely contributes to sensitizing p65-deficient keratinocytes to DNA damage induced death.

Referee #3 for EMM-2013-03541:

Remarks:

The study by Kim and Pasparakis investigates the role of in keratinocyte p65 in the development of skin tumors after DMBA/TPA treatment. The authors found that keratinocyte-specific p65 knock-out inhibits tumorigenesis and inflammatory skin hyperplasia. This is in accordance with a role of NF-kappaB in promoting the survival of mutagenized cells and in enhancing tumor progression by establishing a pro-inflammatory environment. The study is generally well performed and the manuscript is well organized. However, the interpretation of some experiments should be supported by more quantitative data. The paper could be improved by addressing following concerns:

1. The selection of analyzed NF-kappaB-inducible anti-apoptotic genes is not clear. There are several other genes that could be tested, Tnfaip3 and Bcl2a1a for example. Both are highly NF-kappaB inducible and anti-apoptotic.

We thank the reviewer for the useful suggestion to analyze the expression of additional NF-kB dependent survival genes. Following the reviewer's suggestion, we analysed more anti-apoptotic genes such as *Birc5, Bcl2a, and Tnfaip3* (Figure S5). Indeed, the expression of *Tnfaip3*, one of the genes that the reviewer proposed, was impaired in KO in response to DOX, but not to MMS. Accordingly, we have included this information to the main text of the manuscript.

The fact that cIAP2 rescues cell survival after DOX treatment cannot be used as an indication that cIAP2 has a prominent role in vivo.

We would agree with the reviewer that this experiment does not provide experimental proof that reduced cIAP2 expression is the only mechanism sensitizing p65-deficient keratinocytes to DNA damage-induced death but we believe our results do suggest a role of cIAP2 contributing to the phenotype. The transfection experiment, although rather crude, provided further support of our hypothesis that the reduced cIAP2 expression could contribute to the sensitization of p65-deficient keratinocytes to DNA damage induced death. We do not suggest that the lower cIAP2 expression is the sole mechanism sensitizing p65 knockout keratinocytes to DNA damage induced death and we would not expect depletion of cIAP2 in wt keratinocytes to phenocopy p65 deficiency. However, based on the well-established pro-survival role of cIAP2, we believe that our results do support our conclusion that reduced cIAP2 expression is likely to contribute to the inceased sensitivity of p65 knockout keratinocytes to DNA damage induced death.

Furthermore, in Fig 4C, tubulin expression seems to be lower in p65EKO mice. How does that impact the interpretation of the blots? Quantification and normalization of the signal would be helpful.

We appreciate reviewer's insightful comment regarding cIAP2. As we responded to reviewer1, during discussion with other scientists studying cIAPs, we learned that there is no reliable antibody detecting murine cIAP2. In order to address the specificity of the antibody against cIAP2 that we used, we performed a control experiment utilizing cIAP2 KO MEFs and LBW242, a smac mimetic that induces degradation cIAP1 and cIAP2.

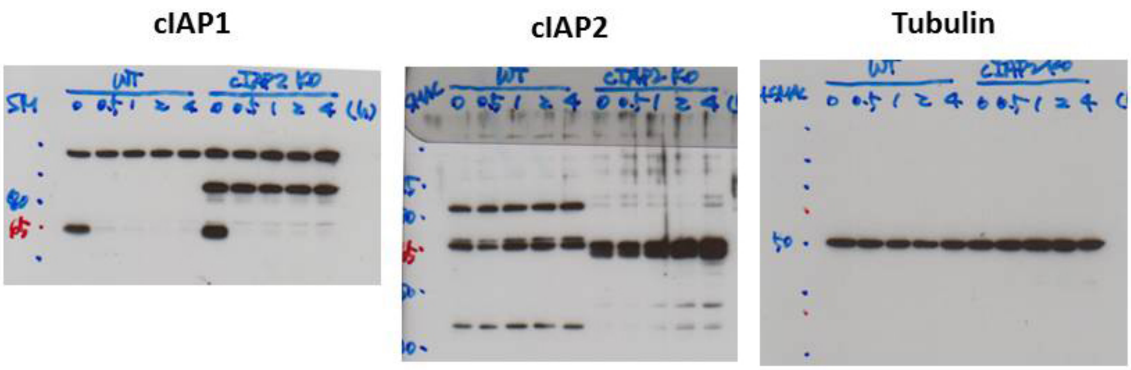

As shown in the original version of our manuscript with keratinocytes, the cIAP2 antibody detected a band at the expected size of cIAP2 (slightly above ~65kDa) in WT MEFs. However, the very same band also appeared in cIAP2 KO MEFs. Although we also see that the antibody detects another slightly larger protein only in WT MEFs, because it is not affected by SM, we are not sure about the identity of the band. As shown in cIAP1 panel, SM efficiently targeted cIAP1 (~65kDa) in both WT and cIAP2 MEFs within 30 min, indicating SM treatment was successful. Since our

control experiment failed to confirm that the antibody specifically recognizes cIAP2, we regrettably decided to remove this figure from the manuscript.

2. *One major questions remains whether the findings are model specific or could be laso observed in skin carcinogenesis models where the inflammatory component is not so prominent as in the DMBA/TPA model.*

It could be. However, what we would like to report here is that p65 knockout in keratinocytes in this model protects mice against tumour development. We believe our *in vivo* data unequivocally demonstrate that p65 expression in keratinocytes is essential for DMBA/TPA-induced tumour development. Whether p65 deficiency might have a different effect in another tumour model remains to be investigated, but we believe such studies are outside the scope of the current manuscript.

3. *Fig 5. Skin hyperplasia should be measured and presented as average epidermal thickness or number of nucleated interfollicular cell layers in all experimental groups.*

We showed representative images as is usual in the vast majority of such studies. In response to the reviewer's comment, we now present quantification of epidermal thickness in these animals in figure 5B of the revised manuscript.

4. *The strain and gender of mice used in the study are not indicated. Authors should also disclose randomization and blinding protocols.*

We'd be happy to include information on the gender of the mice (see table below). The mice used were in C57Bl/6 genetic background (the p65floxed allele was generated using C57Bl/6 ES cells as described in the reference cited for the generation of these animals) and the K14Cre line has been backcrossed to C57Bl/6 for more than 20 generations. Mice were genotyped at weaning age to determine the number of $p65^{EKO}$, $p65^{FL/FL}$ and $p65^{EHET}$ animals in the groups. Then the animals were exposed to DMBA/TPA and tumour development was monitored weekly by an investigator not knowing the genotype of the mice.

	$p65^{FL}$		$p65^{EKO}$		$p65^{EHT}$	
Sex	F	M	F	M	F	M
Total	14	7	15	6	5	5

References

Dajee M, Lazarov M, Zhang JY, Cai T, Green CL, Russell AJ, Marinkovich MP, Tao S, Lin Q, Kubo Y & Khavari PA (2003) NF-kappaB blockade and oncogenic Ras

trigger invasive human epidermal neoplasia. *Nature* **421:** 639–643

Johansen C, Vestergaard C, Kragballe K, Kollias G, Gaestel M & Iversen L (2009) MK2 regulates the early stages of skin tumor promotion. *Carcinogenesis* **30:** 2100–2108

Rebholz B, Haase I, Eckelt B, Paxian S, Flaig MJ, Ghoreschi K, Nedospasov SA, Mailhammer R, Debey-Pascher S, Schultze JL, Weindl G, Förster I, Huss R, Stratis A, Ruzicka T, Röcken M, Pfeffer K, Schmid RM & Rupec RA (2007) Crosstalk between Keratinocytes and Adaptive Immune Cells in an IκBα Protein-Mediated Inflammatory Disease of the Skin. *Immunity* **27:** 296–307

Ryan KM, Ernst MK, Rice NR & Vousden KH (2000) Role of NF-kappaB in p53-mediated programmed cell death. *Nature* **404:** 892–897

Tergaonkar V, Pando M, Vafa O, Wahl G & Verma I (2002) p53 stabilization is decreased upon NFkappaB activation: a role for NFkappaB in acquisition of resistance to chemotherapy. *Cancer Cell* **1:** 493–503

Zhang JY, Green CL, Tao S & Khavari PA (2004) NF-kappaB RelA opposes epidermal proliferation driven by TNFR1 and JNK. *Genes Dev* **18:** 17–22

2nd Editorial Decision 13 May 2014

Thank you for the submission of your manuscript to EMBO Molecular Medicine.

We have now heard back from the three Reviewers whom we asked to re-evaluate your manuscript.

You will see that while Reviewers 3 is now supportive, Reviewers 1 and 2 are still not satisfied that all issues raised were adequately addressed. My impression is that two orders of problems remain.

One is more specific and refers to the cIAP2 protein issue. All three Reviewers mention this as a persisting problem that must be properly solved. Reviewer 1 maintains that fig. 4B cannot stand without proper assessment of protein levels. Reviewer 2 appears to be on the same wavelength and laments the fact that no real effort appears to have been made to address this experimentaly (or even to rebut). The more positive Reviewer 3 also notes that the cIAP2 issue could have been addressed better. Reviewer 2 also notes additional experimental issues that should be addressed.

The other issue is of a more general nature and refers to the over-interpretation /over-statement of findings and conclusions based on the data available. I note that very few changes have been made to the Results and almost none to the Discussion in this revision.

The Reviewers are experts and my impression is that they have been fair and also accepted your argumentation that the "onus to clarify the discrepancies with previous results" is not yours.

As you know, it is EMBO Molecular Medicine policy to allow a single round of revision only. We have now re-discussed your manuscript and are prepared in this case to allow you to submit a re-revised version in the light of these comments. We do agree in fact, that the Reviewers' points are well-taken and thus encourage you to make substantial efforts to improve your mansucript along the lines mentioned. I believe that ultimately this would consolidate your findings and increase the value of your work. Provided this is done comprehensively, I am prepared to make an editorial decision on your manuscript.

I look forward to seeing a revised form of your manuscript as soon as possible.

***** Reviewer's comments *****

Referee #1 (Remarks):

In this manuscript, Kim and Pasparakis used a very well genetically defined model of ablating p65/RelA in the murine keratinocytes (p65EKO mice) they demonstrated that this protein is required for successful papilloma formation in the DMBA-TPA model of skin carcinogenesis. Inability of damaged cells to survive the DMBA assault along with poor inflammatory effects of TPA-mediated tumor promotion in p65EKO mice were proposed as the mechanisms underlying inefficient carcinogenesis in these animals.

In the revised manuscript, authors have addressed several of previous reviewers' comments and reasoned against other comments. Overall, the changes made have to some extent strengthened the manuscript. However, the writing still can be improved and adjusted to avoid over-interpretation of Additional changes can be considered to further improve this work:

1. Page 6: "Therefore, p65 signaling in epidermal keratinocytes was essential for DMBA/TPA-induced skin tumourigenesis". There are two issues with this sentence. First, its meaning is unclear as p65 is a component of transcription factor. Do authors mean "signaling to p65" or "p65 transcriptional activities"? Second, a few small tumors have been seen in p65EKO mice - that means p65 is not essential but required for efficient tumorigenesis.
2. The results shown in Figure 4B cannot be unequivocally interpreted without a crucial control - assessment of the cIAP2 protein level
3. Page 11: Data in Figure 6 are over-interpreted - "These findings suggest that TPA-induced myeloid cell infiltration in the skin depends on p65-dependent expression of inflammatory mediators from epidermal keratinocytes". Actually the data shown only demonstrate that TPA-

induced myeloid infiltration correlates with expression of inflammatory mediators and both of these phenomena depends on p65 activities (whether it is stimulating of expression of inflammatory cytokines or other effects). Similarly, the dependence of TPA-induced hyperplastic response on analyzed chemokines and cytokines have not been investigated (using additional transgenic models or even neutralizing antibodies) to justify the conclusion in the last sentence of the Results.

Referee #2 (Remarks):

In the revised version of the paper Kim and Pasparakis have addressed the various points of concern, mostly by discussing these points in the frame of the rebuttal letter. Experimentally, the number of Langerhans cells was determined and attempts were made to measure cIAP2 levels by western Blot.

I can agree with the arguments of the authors; however I still have major concerns on the interpretation on the function of cIAP2 in the context of the observed phenotype of the mice. The authors still claim that "...reduced cIAP2 expression likely contributes to the sensitization of p65-deficient keratinocytes to genotoxic stress-induced death" (p.12):

- no efforts were made to get experimental proof for this assumption, e.g. via knockdown of Birc3 (as suggested previously but not addressed at all, even in the point by point response), or including the LBW242 (SM) inhibitor, which was used by the authors to determine/disprove the specificity of the cIAP2 antibody.
- observing a rescue from apoptosis in response to Dox upon overexpression of cIAP2 is nice; however, as the authors have no idea about the levels of exogenously expressed protein (or at least mRNA), the significance of this result is limited. Unfortunately, the authors did not comment on transfection efficiency of primary keratinocytes (as requested). I assume that by far not cells were targeted and measuring an almost complete rescue in the frame of a large proportion of non-transfected cells appears remarkable. On the other hand, high overexpression of any protein (which greatly exceed the level of endogenous protein in wildtype cells) involved in cell survival pathways may yield similar results. Combining information on transfection efficiency and measuring total Birc3 mRNA (both endogenously and exogenously expressed) levels may give a better insight into this issue.
- The authors state that "Birc3.... expression was consistently down-regulated in p63-deficient keratinocytes in response to both DOX and MMS...." (p. 9, bottom). This is clearly wrong! Basal level is lower in mutant cells compared to mutant cells; expression is downregulated ONLY in wild-type cells but not in mutant cells; downregulation is ONLY seen in response to DOX but NOT in response to MMS (Fig. 4A). These data (lack of downregulation by MMS) question the role of cIAP2 in the MMS response and clearly ask for a rescue experiment (Birc3 overexpression) in mutant cells in response to MMS.
- It is interesting to note that differences in viability (Fig. 2C) are only seen at single dose of DOX (0,2 M) and MMS (0,2 mM). Differences at 10-fold lower concentration (0.02 mM) are not significant; at 10-fold higher doses viability is similarly low. No information is provided on the concentration of DOX and MMS in Fig. 3 and Fig. 4A (I assume 0,2 M DOX and 0,2 mM MMS was used). Does a lower concentration of either DOX or MMS induce a cellular stress response as measured in Fig. 3B, C and Fig. 4A?

Referee #3 (Remarks):

The authors have improved the manuscript. The inability to detect cIAP on the protein level deters somewhat from the impact but does not overly affect the results. One would expect that authors show at least cIAP expression levels in transfected keratinocytes using a tag antibody.

2nd Revision - authors' response 16 May 2014

Referee #1 (Remarks):

In this manuscript, Kim and Pasparakis used a very well genetically defined model of ablating p65/RelA in the murine keratinocytes (p65EKO mice) they demonstrated that this protein is required for successful papilloma formation in the DMBA-TPA model of skin carcinogenesis. Inability of damaged cells to survive the DMBA assault along with poor inflammatory effects of TPA-mediated tumor promotion in p65EKO mice were proposed as the mechanisms underlying inefficient carcinogenesis in these animals.

In the revised manuscript, authors have addressed several of previous reviewers' comments and reasoned against other comments. Overall, the changes made have to some extent strengthened the manuscript. However, the writing still can be improved and adjusted to avoid over-interpretation of

Additional changes can be considered to further improve this work:

1. Page 6: "Therefore, p65 signaling in epidermal keratinocytes was essential for DMBA/TPA-induced skin tumourigenesis". There are two issues with this sentence. First, its meaning is unclear as p65 is a component of transcription factor. Do authors mean "signaling to p65" or "p65 transcriptional activities"? Second, a few small tumors have been seen in p65EKO mice - that means p65 is not essential but required for efficient tumorigenesis.

To address the concern of the reviewer we have modified this sentence in the revised manuscript to: "Therefore, p65-mediated NF-κB activation in epidermal keratinocytes is required for efficient DMBA/TPA-induced skin tumourigenesis".

2. The results shown in Figure 4B cannot be unequivocally interpreted without a crucial control - assessment of the cIAP2 protein level

Unfortunately due to the lack of antibodies detecting mouse cIAP2 we cannot assess cIAP2 protein levels. We agree with the reviewer that without assessment of protein expression levels of cIAP2 this experiment is difficult to interpret. For this reason and following the comments of the other reviewers, we have decided to remove this figure from the paper and base our suggestion that reduced cIAP2 expression could contribute to the sensitization of p65-deficient keratinocytes to DNA damage induced apoptosis only on our results on mRNA expression levels.

3. Page 11: Data in Figure 6 are over-interpreted - "These findings suggest that TPA-induced myeloid cell infiltration in the skin depends on p65-dependent expression of inflammatory mediators from epidermal keratinocytes". Actually the data shown only demonstrate that TPA-induced myeloid infiltration correlates with expression of inflammatory mediators and both of these phenomena depends on p65 activities (whether it is stimulating of expression of inflammatory cytokines or other effects). Similarly, the dependence of TPA-induced hyperplastic response on analyzed chemokines and cytokines have not been investigated (using additional transgenic models or even neutralizing antibodies) to justify the conclusion in the last sentence of the Results.

To address the reviewers' concerns we have modified this sentence in the revised manuscript to: "Therefore, epidermal keratinocyte-specific ablation of p65 inhibited the TPA-induced expression of chemokines and cytokines and the tumour-promoting inflammatory hyperplastic response in the skin".

Referee #2 (Remarks):

In the revised version of the paper Kim and Pasparakis have addressed the various points of concern, mostly by discussing these points in the frame of the rebuttal letter. Experimentally, the number of Langerhans cells was determined and attempts were made to measure cIAP2 levels by western Blot.

I can agree with the arguments of the authors; however I still have major concerns on the interpretation on the function of cIAP2 in the context of the observed phenotype of the mice. The authors still claim that "...reduced cIAP2 expression likely contributes to the sensitization of p65-deficient keratinocytes to genotoxic stress-induced death" (p.12):

- no efforts were made to get experimental proof for this assumption, e.g. via knockdown of Birc3 (as suggested previously but not addressed at all, even in the point by point response), or including the LBW242 (SM) inhibitor, which was used by the authors to determine/disprove the specificity of the cIAP2 antibody.

As discussed above, there are no antibodies that detect mouse cIAP2 therefore it is impossible to address cIAP2 protein levels in the cells of our mice. Because of this problem, also knockdown experiments would be meaningless as it would be impossible to assess the efficiency of the knockdown on protein levels. Using smac-mimetic compounds would also not be an option as these compounds result in the degradation of both cIAP1 and cIAP2, therefore any effect could not be attributed specifically to cIAP2.

- observing a rescue from apoptosis in response to Dox upon overexpression of cIAP2 is nice; however, as the authors have no idea about the levels of exogenously expressed protein (or at least mRNA), the significance of this result is limited. Unfortunately, the authors did not comment on transfection efficiency of primary keratinocytes (as requested). I assume that by far not cells were targeted and measuring an almost complete rescue in the frame of a large proportion of non-transfected cells appears remarkable. On the other hand, high overexpression of any protein (which greatly exceed the level of endogenous protein in wildtype cells) involved in cell survival pathways may yield similar results. Combining information on transfection efficiency and measuring total Birc3 mRNA (both endogenously and exogenously expressed) levels may give a better insight into this issue.

We agree with the reviewer that without assessment of protein expression levels of cIAP2 this experiment is difficult to interpret. For this reason and following the comments of the other reviewers, we have decided to remove this figure from the paper and base our suggestion that reduced cIAP2 expression could contribute to the sensitization of p65-deficient keratinocytes to DNA damage induced apoptosis only on our results on mRNA expression levels.

- The authors state that "Birc3.... expression was consistently down-regulated in p63-deficient keratinocytes in response to both DOX and MMS...." (p. 9, bottom). This is clearly wrong! Basal level is lower in mutant cells compared to mutant cells; expression is downregulated ONLY in wild-type cells but not in mutant cells; downregulation is ONLY seen in response to DOX but NOT in response to MMS (Fig. 4A). These data (lack of downregulation by MMS) question the role of cIAP2 in the MMS response and clearly ask for a rescue experiment (Birc3 overexpression) in mutant cells in response to MMS.

We meant to write that Birc3 expression in p65-deficient cells is decreased both at basal levels and also after DNA damage, as shown in figure 4A. This part of the text has now been removed from the manuscript.

- It is interesting to note that differences in viability (Fig. 2C) are only seen at single dose of DOX (0,2 μM) and MMS (0,2 mM). Differences at 10-fold lower concentration (0.02 mM) are not significant; at 10-fold higher doses viability is similarly low. No information is provided on the concentration of DOX and MMS in Fig. 3 and Fig. 4A (I assume 0,2 μM DOX and 0,2 mM MMS was used). Does a lower concentration of either DOX or MMS induce a cellular stress response as measured in Fig. 3B, C and Fig. 4A?

The experiments in Figures 3 and 4A were performed using 0.5 μM DOX and 0.5 mM MMS. This information has now been included in the figure legends. Regarding the observation that at 10x lower and at 10x higher concentration the differences between wt and knockout cells are not significant, this is not unusual in such experiments. P65-dependent signalling contributes to the response of cells to DNA damage but is by far not the only pathway determining if the cells will die or survive after DNA damage. Usually the responses of biological systems are not black and white therefore it is rather the norm that differences are observed within a certain range of the response. Here we determined the optimal concentration of the genotoxic agents and used this concentration throughout our experiments.

Referee #3 (Remarks):

The authors have improved the manuscript. The inability to detect cIAP on the protein level deters somewhat from the impact but does not overly affect the results. One would expect that authors show at least cIAP expression levels in transfected keratinocytes using a tag antibody.

We agree with the reviewer that without assessment of protein expression levels of cIAP2 this experiment is difficult to interpret. For this reason and following the comments of the other reviewers, we have decided to remove this figure from the paper and base our suggestion that reduced cIAP2 expression could contribute to the sensitization of p65-deficient keratinocytes to DNA damage induced apoptosis only on our results on mRNA expression levels.

3rd Editorial Decision 19 May 2014

Thank you for the submission of your revised manuscript to EMBO Molecular Medicine. I am pleased to inform you that we will be able to accept your manuscript pending the following final amendments:

1) As per our Author Guidelines, the description of all reported data that includes statistical testing must state the name of the statistical test used to generate error bars and P values, the number (n) of independent experiments underlying each data point (not replicate measures of one sample), and the actual P value for each test (not merely 'significant' or 'P < 0.05').

2) Please modify your manuscript to abide by the following (from the Guide to Authors): "For Research Articles and Reports submitted to EMBO Molecular Medicine reporting experiments on live vertebrates and/or higher invertebrates, the corresponding author must confirm that all experiments were performed in accordance with relevant guidelines and regulations. The manuscript must include a statement in the Materials and Methods identifying the institutional and/or licensing

committee approving the experiments, including any relevant details."

3) We are now encouraging the publication of source data, particularly for electrophoretic gels and blots, with the aim of making primary data more accessible and transparent to the reader. Would you be willing to provide a PDF file per figure that contains the original, uncropped and unprocessed scans of all or at least the key gels used in the manuscript? The PDF files should be labeled with the appropriate figure/panel number, and should have molecular weight markers; further annotation may be useful but is not essential. The PDF files will be published online with the article as supplementary "Source Data" files. If you have any questions regarding this just contact me.

4) In your point-by-point-response to the Reviewers you included a number of figures. Would you please confirm whether or not we could publish these figures as part of the peer review process file?

I look forward to receiving your final revised version as soon as possible and in any case, possibly within two weeks

© EMBO
